# Misregulation of Scm3p/HJURP Causes Chromosome Instability in *Saccharomyces cerevisiae* and Human Cells

**DOI:** 10.1371/journal.pgen.1002303

**Published:** 2011-09-29

**Authors:** Prashant K. Mishra, Wei-Chun Au, John S. Choy, P. Henning Kuich, Richard E. Baker, Daniel R. Foltz, Munira A. Basrai

**Affiliations:** 1 Genetics Branch, Center for Cancer Research, National Cancer Institute, National Institutes of Health, Bethesda, Maryland, United States of America; 2 Department of Biochemistry and Molecular Genetics, University of Virginia, Charlottesville, Virginia, United States of America; 3 Department of Molecular Genetics and Microbiology, University of Massachusetts Medical School, Worcester, Massachusetts, United States of America; Duke University, United States of America

## Abstract

The kinetochore (centromeric DNA and associated proteins) is a key determinant for high fidelity chromosome transmission. Evolutionarily conserved Scm3p is an essential component of centromeric chromatin and is required for assembly and function of kinetochores in humans, fission yeast, and budding yeast. Overexpression of *HJURP*, the mammalian homolog of budding yeast Scm3p, has been observed in lung and breast cancers and is associated with poor prognosis; however, the physiological relevance of these observations is not well understood. We overexpressed *SCM3* and *HJURP* in *Saccharomyces cerevisiae* and *HJURP* in human cells and defined domains within Scm3p that mediate its chromosome loss phenotype. Our results showed that the overexpression of *SCM3* (*GALSCM3*) or *HJURP* (*GALHJURP*) caused chromosome loss in a wild-type yeast strain, and overexpression of *HJURP* led to mitotic defects in human cells. *GALSCM3* resulted in reduced viability in kinetochore mutants, premature separation of sister chromatids, and reduction in Cse4p and histone H4 at centromeres. Overexpression of *CSE4* or histone H4 suppressed chromosome loss and restored levels of Cse4p at centromeres in *GALSCM3* strains. Using mutant alleles of *scm3*, we identified a domain in the N-terminus of Scm3p that mediates its interaction with *CEN* DNA and determined that the chromosome loss phenotype of *GALSCM3* is due to centromeric association of Scm3p devoid of Cse4p/H4. Furthermore, we determined that similar to other systems the centromeric association of Scm3p is cell cycle regulated. Our results show that altered stoichiometry of Scm3p/HJURP, Cse4p, and histone H4 lead to defects in chromosome segregation. We conclude that stringent regulation of *HJURP* and *SCM3* expression are critical for genome stability.

## Author Summary

Proper chromosome segregation is essential for normal cell proliferation. Segregation errors lead to aneuploidy, a direct cause of birth defects and a hallmark of cancer. The kinetochore (centromeric DNA and associated proteins) is one of the key determinants for faithful chromosome transmission. Misregulation of kinetochore proteins such as *HJURP* has been observed in various cancers, however the biological relevance of this observation is not well understood. We determined that altered dosage of *HJURP* and its budding yeast homolog *SCM3* leads to defects in chromosome segregation in yeast and human cells. We identified the centromeric DNA–interacting domain of Scm3p and determined that association of Scm3p devoid of Cse4p leads to chromosome segregation defects. Our findings suggest that stringent regulation of Scm3p/*HJURP*, Cse4p, and histone H4 is critical for maintenance of genome stability.

## Introduction

Proper chromosome segregation is essential for normal cell proliferation. Errors in this process lead to birth defects, developmental disorders, aneuploidy and possibly cancer [1]. The kinetochore (centromeric DNA and associated proteins), microtubules, spindle pole bodies, condensins, and telomeres, as well as regulatory components that establish checkpoints [2] are essential for faithful chromosome segregation. The centromere (*CEN*) is the *cis-*acting DNA locus that specifies the site of kinetochore assembly, participates in the attachment of chromosomes to the mitotic and meiotic spindles and maintains cohesion between sister chromatids [3], [4]. *CEN* DNA sequences are highly variable among eukaryotes. Budding yeast contains “point” centromeres, whereas other eukaryotes, for example fission yeast, fruit fly, plants and mammals have “regional” centromeres. The point centromeres are small (∼125 bp in size) and consist of three conserved DNA elements (CDEI, CDEII, and CDEIII), whereas regional centromeres are relatively large in size (40–4000 kb) and contain species-specific arrays of repeated DNA [5]–[7]. Despite *CEN* DNA sequence variation, replacement of histone H3 in centromeric chromatin by the centromere-specific histone H3 variant CenH3 is universally conserved [6]. CenH3 homologs (Cse4p in budding yeast, Cnp1 in fission yeast, CID in fruit fly, HTR12 in Arabidopsis and *CENP-A* in humans) function as an epigenetic mark in all organisms and is essential for determining centromere identity and proper kinetochore function [8]–[14]. In budding yeast, kinetochore sub-complexes Ctf3p (Ctf3p, Mcm16p and Mcm22p) and COMA (Ame1p, Ctf19p, Mcm21p and Okp1p) exhibit genetic and physical interactions with Cse4p [15], [16].

It has been shown that the point centromeres of *Saccharomyces cerevisiae* consist of a single Cse4p nucleosome [17], and a novel inner kinetochore protein Scm3p (*S*uppressor of *C*hromosome *M*issegregation) is required for the centromeric deposition of Cse4p [18]–[23]. Scm3p is evolutionarily conserved with homologs identified in a range of species including fission yeast (Sp-Scm3p), fungi, humans (*HJURP*, Holliday Junction Recognition Protein), and other vertebrates [24], [25]. *HJURP* and Scm3p share a common ancestry and both proteins have an evolutionarily conserved N-terminal domain [24]. This evolutionary conserved domain of *HJURP* directly interacts with the centromere targeting domain (CATD) of *CENP-A* [25]–[27]. In budding yeast, depletion of Scm3p leads to cell cycle arrest and chromosome segregation defects [18]–[20], [28], [29]. Studies from fission yeast have shown that Sp-Scm3p functions as a Cnp1p receptor and facilitates its assembly into centromeric chromatin [30], [31]. Sp-Scm3p shows cell cycle dependent centromeric enrichment, and it dissociates from centromeres in early mitosis [30], [31]. Similarly, studies with human cells have shown that centromeric association of *HJURP* is cell cycle regulated and *HJURP* promotes the deposition and maintenance of *CENP-A* at centromeres [26], [32], [33].

Previous studies have shown that balanced stoichiometry of histones and CenH3 is important for chromosome transmission fidelity. For example, maintenance of a balanced ratio between Cse4p/Cnp1, histone H4 or histone H3 affects centromere function in fission and budding yeast, and altered dosage of these proteins results in chromosome missegregation [34], [35]. In other systems such as, fruit fly, CID overexpression results in its mislocalization, formation of ectopic kinetochores and aneuploidy [36]. Altered expression and mis-localization of *CENP-A* has been observed in colorectal tumors [37]. Also, altered dosage and mis-localization of kinetochore proteins, such as *CENP-H*, *Aurora-B* and *INCENP* has been observed in various types of tumor cells [37]–[39]. As observed for *CENP-A*, the expression of its chaperone, *HJURP* is also tightly regulated and this is critical for high fidelity chromosome transmission, because perturbation of its expression leads to mitotic defects [32], [38]. Overexpression and mis-localization of *HJURP* has been observed in lung cancer cell lines and these observations were linked with chromosomal instability and immortality of cancer cells [38]. Notably, overexpression of *HJURP* has also been observed in breast cancer cells, where patients with elevated *HJURP* mRNA levels showed decreased survival rate and were more sensitive to radiotherapy [39]. However, the physiological basis for these observations is not well understood, nor is it known if *HJURP* overexpression plays a direct role in cancer initiation or progression. Considering the structural and functional homology between *SCM3* and *HJURP*, we used budding yeast to investigate the physiological consequences of *SCM3/HJURP* overexpression and understand how missregulation of these proteins affects chromosome segregation.

Here, we show that overexpression of *SCM3* leads to defects in chromosome transmission fidelity in a wild-type strain, reduced viability in kinetochore mutants, premature separation of sister chromatids, perturbation of its centromeric enrichment pattern and reduced levels of Cse4p at centromeres. Overexpression of *CSE4* (*GALCSE4*) or histone H4 (*GALH4*) suppressed the *GALSCM3*-induced chromosome loss and restored levels of Cse4p at centromeres. Our results revealed that the N-terminus of Scm3p (first 100 amino acids) mediates its centromeric interaction and established that Scm3p can associate with centromeres independent of Cse4p. Using mutant alleles of *scm3*, we determined that the kinetochore integrity defects of *GALSCM3* strains are due to centromeric association of Scm3p devoid of Cse4p. Our results show that overexpression of *HJURP* in budding yeast, and in human cells leads to chromosome loss.

## Results

### Misregulation of *SCM3* leads to defects in chromosome transmission fidelity (*ctf*) in wild-type strains and reduced levels of Cse4p at *CEN* DNA

**Figure 1:**
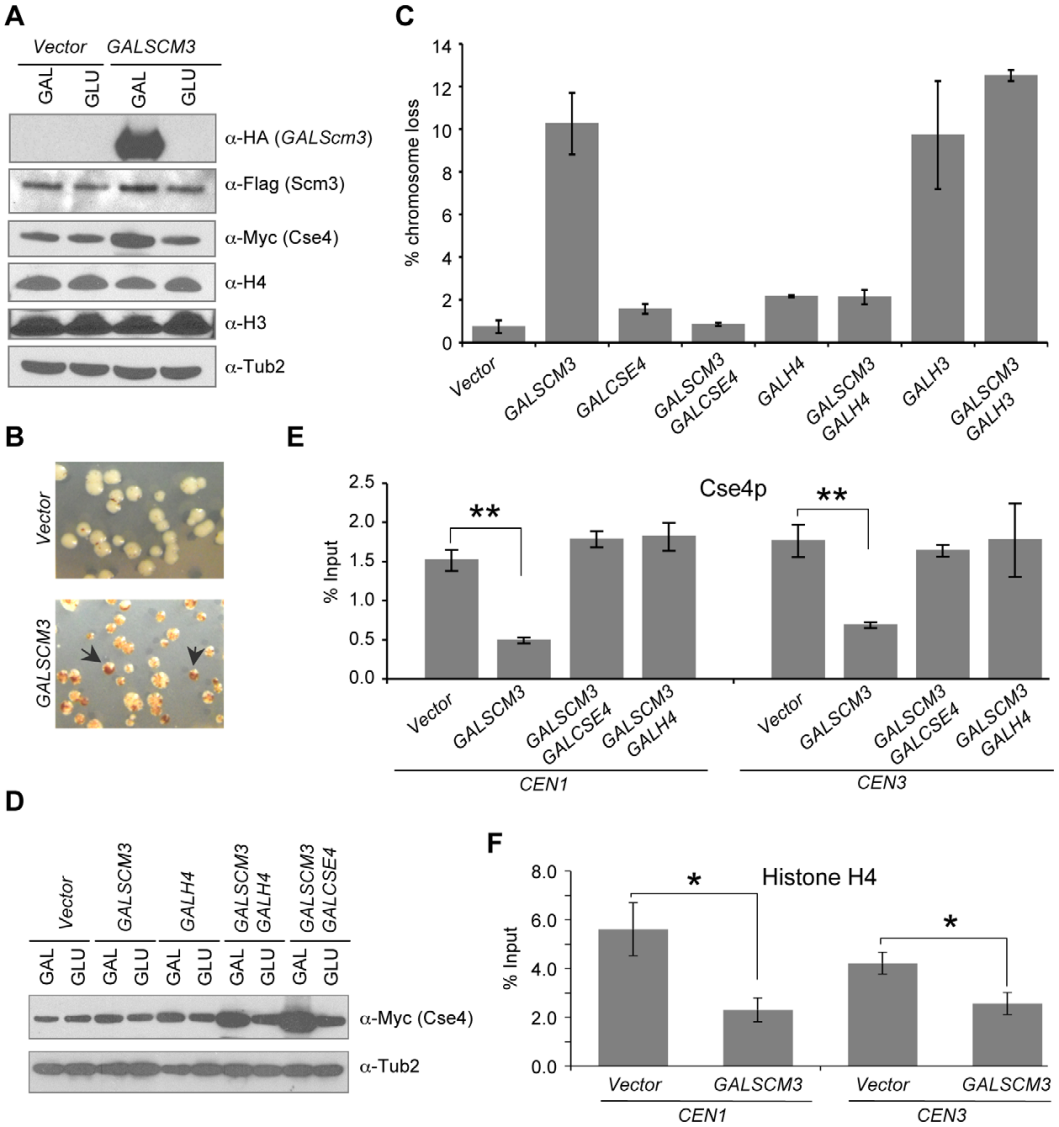
Overexpression of *SCM3* causes chromosome instability and reduction in *CEN*-bound Cse4p and histone H4. (A) Western blot analysis was done using whole cell protein extracts prepared from wild type strains (RC154 or JG595) transformed with *GALSCM3HA* (pMB1306), or vector (pRS426 *GAL1*). Strains were grown to logarithmic phase of growth in synthetic media and gene expression induced in the presence of galactose (2%) at 30°C for 12 hours. Blots were probed with anti-HA for expression of *GALSCM3HA*, anti-FLAG for expression of FLAG tagged Scm3p expressed from its own promoter, anti-Myc for expression of Cse4p, anti-H3 (abcam # ab1791-100) for expression of histone H3, anti-H4 (Millipore # 62-141-13) for expression of histone H4 and α-Tub2p served as a loading control. (B) Wild type strains with reporter chromosome fragment (YPH1018) expressing *SCM3* from *GAL1* promoter (pMB1306) or vector (pRS426 *GAL1*) were plated on SC-URA with limiting adenine and galactose (2%). Representative images showing red sectors in white colonies represent the loss of the non-essential chromosome fragment (CF). Arrows indicate colonies that show a half red-half white phenotype which represent CF loss in the first cell division. (C) *GALSCM3-*induces chromosome missegregation, which is suppressed by *GALCSE4* and *GALH4*. Quantification of chromosome loss by half-sector analysis. Reporter strains with chromosome fragment (YPH1018) transformed with vectors (pRS425 *GAL1*, pRS426 *GAL1*), *GALSCM3HA* (pMB1306), *GALCSE4* (pMB1147), *GALSCM3HA*, *GALCSE4* (pMB1306, pMB1147), *GALH4* (pMB1158), *GALSCM3HA*, *GALH4* (pMB1306, pMB1158), *GALH3* (pMB1159), and *GALSCM3HA*, *GALH3* (pMB1306, pMB1159) were assayed for chromosome loss. At least 3000 colonies were counted and values represent the average ± standard error for three independent transformants normalized to the value of 100. (D) Co-overexpression of histone *H4* and *SCM3* increases the levels of Cse4p. Western blot analysis was done using whole cell protein extracts prepared from a wild type strain (JG595) transformed with vector (pRS425 *GAL1*), *GALSCM3* (pMB1193), GALH4 (pMB1158), *GALSCM3*, *GALH4* (pMB1193, pMB1158), and *GALSCM3*, *GALCSE4* (pMB1193, pMB976). Strains were grown to logarithmic phase of growth in media selective for the plasmid and gene expression induced in the presence of galactose (2%) at 30°C for 12 hours. Blots were probed with anti-Myc for expression of Myc tagged Cse4p expressed from its own promoter and α-Tub2p served as a loading control. (E) *GALSCM3* strains show reduced levels of *CEN*-associated Cse4p. ChIP experiments were done using wild type strain expressing Cse4p-Myc from its endogenous promoter (YMB6094) with vectors (pRS425 *GAL1*, pRS426 *GAL1*), *GALSCM3HA* (pMB1306), *GALSCM3HA*, *GALCSE4* (pMB1306, pMB976), and *GALSCM3HA*, *GALH4* (pMB1306, pMB1158) grown in galactose (2%) media for 12 hours and immunoprecipitation were done with α-Myc, and α-GST (mock) antibodies. Enrichment of Cse4p to *CEN1* and *CEN3* DNA was determined by qPCR and is shown as % input. Average from at least three independent experiments ± standard error is shown. *p value <0.05, **p value <0.01, Student's t test. (F) *GALSCM3* strains show reduced levels of *CEN*-associated histone H4. ChIP experiments were done using wild type strain (RC154) with vectors (pRS426 *GAL1*), or *GALSCM3HA* (pMB1306) grown in galactose (2%) media for 12 hours and immunoprecipitation were done with α-H4, and α-GST (mock) antibodies. Enrichment of histone H4 to *CEN1* and *CEN3* DNA was determined by qPCR and is shown as % input. Average from at least three independent experiments ± standard error is shown. *p value <0.05, **p value <0.01, Student's t test.

To examine the physiological consequence of misregulation of *SCM3*, we assayed the chromosome loss phenotype of wild-type strains overexpressing *SCM3* from a *GAL1* promoter on a multi-copy plasmid. Western blot analysis confirmed the galactose-induced overexpression of *GALSCM3HA* (Figure 1A). The loss of a non-essential reporter chromosome fragment (CF) in this strain background results in red sectors in an otherwise white colony. We determined the chromosome loss rate by counting the number of colonies that were at least half red on galactose media reflecting the loss of CF in the first cell division (Figure 1B, see arrows). Overexpression of *SCM3* results in a 10-fold higher chromosome loss rate compared to strains expressing vector alone (Figure 1C). The chromosome loss phenotype of the control vector alone strain is similar to that previously reported for wild-type strains on galactose media [35]. Strains expressing untagged *GALSCM3* or *GALSCM3HA* integrated into the genome had chromosome loss rates similar to that of plasmid-borne *GALSCM3HA* strains (Figure S1). We previously reported that overexpression of histone H3 (*GALH3*) alters the stoichiometry between Cse4p and histone H3 leading to defective kinetochores and a chromosome loss phenotype in wild type yeast strains [35]. The chromosome loss phenotype of strains co-expressing *GALSCM3* and *GALH3* is higher but not statistically different than those expressing *GALSCM3* or *GALH3* alone (Figure 1C).

Scm3p directly binds and forms a stoichiometric complex with Cse4p and histone H4, and is required for their assembly into centromeric chromatin [18]–[20]. Similarly, *HJURP* interacts stoichiometrically with CENP-A/H4 [26], [27]. We reasoned that the chromosome loss phenotype of *GALSCM3* strains may be due to imbalanced stoichiometry between Scm3p and Cse4p/H4. Hence, we determined the effect of overexpression of *CSE4* in *GALSCM3* strains. Our results showed that *GALCSE4* suppressed the chromosome loss phenotype of *GALSCM3* strains (Figure 1C). Previous studies have shown that overexpression of histone H4 (*GALH4*) suppresses certain *cse4* alleles [40]. Consistent with these observations, we found that *GALH4* (*GALHHF1*) suppressed the chromosome loss phenotype of *GALSCM3* strains (Figure 1C). We performed western blot analysis to determine if overexpression of Scm3p affects the levels of Cse4p, Histone H3 or Histone H4. We found that *GALSCM3* strains show slightly higher levels of Cse4p, whereas levels of histone H3 or H4 are not affected in these strains (Figure 1A). We also observed higher levels of Cse4p in strains co-expressing *GALSCM3* with either *GALH4* or *GALCSE4* when compared to strains expressing *GALSCM3* or *GALH4* alone (Figure 1D). The increased levels of Cse4p in *GALSCM3* strains are consistent with a recent report describing a role for Scm3p in protecting Cse4p from ubiquitin-mediated degradation [41]. The increased levels of Cse4p in *GALH4* strains can be explained by previous observations for genetic interactions between Cse4p and histone H4 [40].

Based on the suppression of *GALSCM3*-induced chromosome loss phenotype by *GALCSE4* and *GALH4*, we hypothesized that excess Scm3p devoid of Cse4p/H4 may associate with *CEN* DNA. Hence, we performed chromatin immunoprecipitation (ChIP) experiments to measure the levels of *CEN*-bound Cse4p in *SCM3* overexpressing (*GALSCM3*) and vector strains. Consistent with our hypothesis, we observed that *GALSCM3* strains showed about 3-fold reduction in the levels of Cse4p at both *CEN1* (0.54% of input) and *CEN3* (0.44%) compared to vector alone (1.50% at *CEN1*, and 1.48% at *CEN3*) (Figure 1E). *CEN*-bound H4 was also reduced in strains overexpressing *SCM3*. ChIP results showed a 2- to 3-fold reduction in *CEN*-bound histone H4 in *GALSCM3* (2.30% at *CEN1*, and 2.57% at *CEN3*) than vector alone (5.61% at *CEN1*, and 4.22% at *CEN3*) strains (Figure 1F). Western blot analysis showed that the reduction in *CEN*-bound Cse4p or histone H4 in *GALSCM3* strains was not due to reduced expression of Cse4p or histone H4 (Figure 1A). Furthermore, *CEN*-associated Cse4p in the *GALSCM3* strain was restored to wild-type levels in the presence of *GALCSE4* or *GALH4* (Figure 1E). These results indicate that centromeric association of excess Scm3p devoid of Cse4p/H4 contributes to the chromosome loss phenotype in *GALSCM3* strains.

In order to determine if the chromosome loss phenotype of *GALSCM3* strains may be due to mislocalization of Scm3p and/or Cse4p to non-centromeric regions, we performed ChIP experiments using strains expressing *SCM3* from its native promoter (control) or a *GAL1* promoter. We examined the association of Scm3p and Cse4p at *CEN* DNA, AT rich intergenic regions (*IR1*, *IR2*, *IR3*, and *IR4*), and transcribed regions (*AGP1*, *CWH43*, *ACT1*, and *YGL036W*). The choice of the intergenic regions was based on the rationale that CDEII of *CEN* DNA is also AT rich and we have previously shown association of a mutant form of Cse4p (Cse4K16R) when overexpressed at two of these regions (*IR1*, *IR2*) [35]. Our results showed higher levels of *GALSCM3* at the *CEN*3 (∼1.5-fold) compared to *SCM3* expressed from its own promoter (Figure S2A). Also, consistent with earlier results (Figure 1E), we observed lower levels of Cse4p at *CEN*3 in *GALSCM3* strains (Figure S2B). We did not see association of either Scm3p or Cse4p at the intergenic (*IR1*, *IR2*, *IR3*, and *IR4*) or transcribed (*AGP1*, *CWH43*, *ACT1*, and *YGL036W*) regions in the control or *GALSCM3* strains (Figure S2A, S2B). Based on these results, we conclude that the chromosome loss phenotype of *GALSCM3* strains is not due to non-centromeric association of Scm3p and Cse4p.

### Overexpression of *SCM3* alters its cell cycle–dependent centromeric association pattern

**Figure 2:**
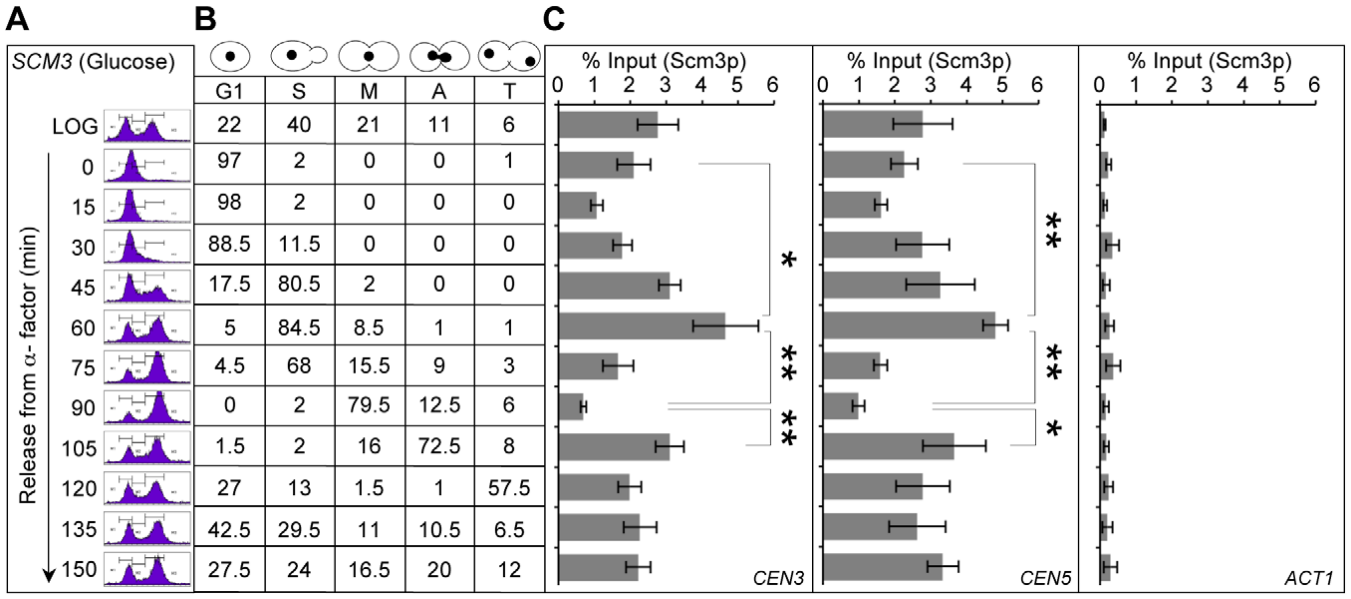
Centromeric association of Scm3p is cell cycle regulated. A wild-type strain (RC154) with FLAG tagged Scm3p expressed from its own endogenous promoter was grown in YPD, synchronized in G1 with α-factor, washed, and released into pheromone-free YPD medium. Samples were taken at time points (min) after release from G1. (A) DNA content was determined by FACS. (B) Cell cycle stages were determined based on cell morphology and nuclear position by microscopic examination of 200 cells for each time point as described in Materials and Methods. (C) Enrichment levels of Scm3p at *CEN3*, *CEN5* and *ACT1* (background control). ChIP experiments were done using α-FLAG (Scm3p), and α-GST (mock) antibodies. The enrichment of Scm3p at *CEN3*, *CEN5*, and *ACT1* was determined by qPCR and is shown as % input. Average from at least three independent experiments ± standard error is shown. *p value <0.05, **p value <0.01 derived from the Student's t test.

Fission yeast Scm3 (Sp-Scm3p) is cell cycle regulated, with Sp-Scm3p dissociating from centromeres in early mitosis [30], [31]. In humans, *HJURP* also exhibits cell cycle-regulated *CEN* localization, showing strong enrichment in G1 and depletion during G2 and mitosis [26], [32]. However, in budding yeast, centromeric localization pattern of Scm3p through the cell cycle has not been determined. Hence, we used ChIP to determine the centromeric localization pattern of FLAG-Scm3p expressed from its native promoter in three independent cell cycle synchronization experiments. In the first experiment, we did ChIP analysis using chromatin from cultures grown in yeast extract-peptone medium with 2% glucose (YPD), synchronized in G1 with alpha factor and released into pheromone free media (Figure 2). Based on nuclear and cell morphology, cells were categorized as G1, S, M (metaphase), A (anaphase) and T (telophase) as described in Materials and Methods [42]. Our results showed that centromeric association of Scm3p is lower in cells released from alpha factor arrest (15 min), maximal in S phase cells (45–60 min), reduced in early mitotic cells (90 min), and enriched in anaphase cells (105 min) (Figure 2). We did not detect Scm3p enrichment at *ACT1* used as a background control (Figure 2C). These results show that centromeric association of Scm3p is cell cycle regulated. We validated these results in two additional experiments using cells arrested with: a) alpha factor and released into medium containing nocodazole (Figure S3A), or b) nocodazole and released into medium containing alpha factor (Figure S3B). Similar to results in Figure 2, we observed a cell cycle regulated pattern of *CEN* association of Scm3p with lower levels in cells released from alpha factor (15–30 min, Figure S3A), maximal in S phase cells (45 min, Figure S3A), reduction in early mitotic cells (75 to 105 min, Figure S3A; and 0 min, Figure S3B) followed by enrichment in anaphase cells (20–30 min, Figure S3B). Western blot analysis showed that expression of Scm3p is not cell cycle regulated (Figure S4). We conclude that the mitotic depletion of centromeric Scm3p in budding yeast is largely similar to that of fission yeast where Sp-Scm3p and to humans where *HJURP* show a transient depletion at the *CEN* during mitosis [26], [30]–[32].

In order to rule out the possibility that our observations for Scm3p may be due to a general of effect of the cell cycle on association of kinetochore proteins to the centromeres, we examined the association of Cse4p at *CEN* DNA through the cell cycle (Figure S5). We observed lower levels of Cse4p in cells released from alpha factor (15 min), higher levels in S phase cells (45 min) and unlike Scm3p, the levels of Cse4p at *CEN* DNA did not show transient depletion in early mitotic cells (75–90 min). The centromeric enrichment of Cse4p in S phase cells is consistent with fluorescence microscopy results demonstrating more intense Cse4-GFP foci in S phase cells [43]. The reduced levels of *CEN*-bound Cse4p at 60 min time point may be due to cells in late S phase and those exiting S phase (Figure S5).

**Figure 3:**
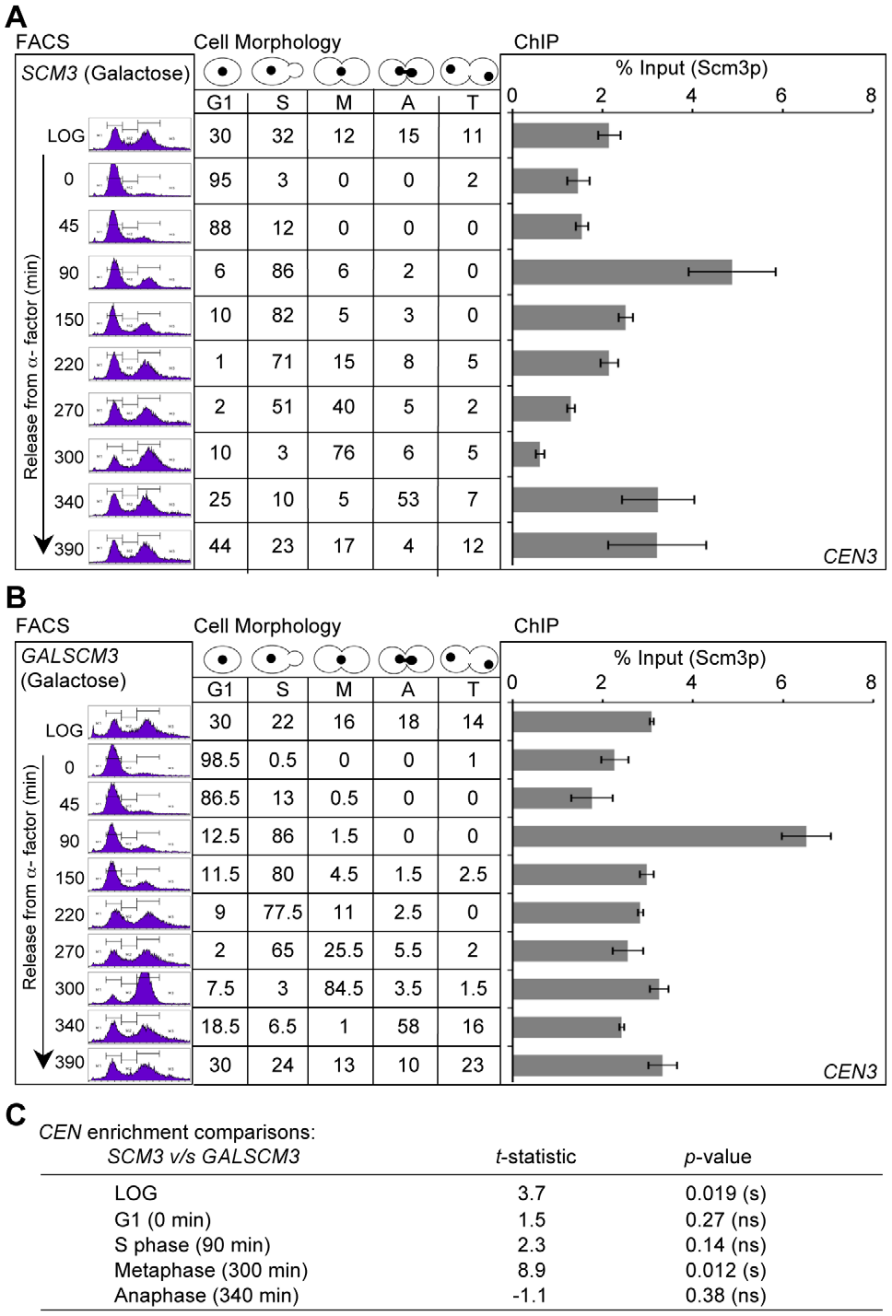
Overexpression of *SCM3* alters its cell cycle–regulated centromeric association pattern. (A) Wild-type strain expressing FLAG-tagged Scm3p (*SCM3*) from its own endogenous promoter (RC154) were grown in minimal media with galactose (2%) to logarithmic phase, treated with α-factor for G1 arrest, washed, and released into pheromone-free media. Samples were taken at time points (min) after release from G1 arrest. DNA content (FACS), Cell cycle stages based on cell shape and nuclear position (cell morphology), and levels of Scm3p at *CEN* DNA (ChIP) were determined as described in Figure 2. (B) Wild-type strain (RC100) overexpressing HA-tagged Scm3p from *GAL1* promoter (*GALSCM3*) was grown in minimal media with galactose (2%), synchronized in G1 with α-factor, washed, and released into pheromone-free media. Samples were taken at time points (min) after release from G1. DNA content (FACS), cell cycle stages (cell morphology) and the levels of Scm3p at *CEN* DNA (ChIP) were determined as described in (A) above. (C) Statistical comparisons of *CEN*-bound Scm3p levels at different cell cycle stages between strains expressing Scm3p from endogenous (panel A above) or *GAL1* promoter (panel B above). Significance was determined using Student's t-test. The t-statistic and *p*-values are shown: significant (s), not significant (ns).

Next, we determined if overexpression of Scm3p affects its cell cycle regulated centromeric association pattern. Thus, we compared the centromeric association pattern of FLAG-Scm3p expressed from its native promoter (control strain *SCM3*, Figure 3A) to the HA-Scm3p expressed from a *GAL1* promoter (*GALSCM3*, Figure 3B). Cultures were grown in synthetic media with 2% galactose, synchronized in G1 with alpha factor and released into pheromone free media. Centromeric enrichment pattern of Scm3p in S phase (90 min) and anaphase (340 min) is similar in strains expressing *SCM3* either from its native (control, Figure 3A) or *GAL1* (*GALSCM3*, Figure 3B) promoter and the difference observed for these time points is not statistically significant (*p*-value >0.05, Figure 3C). However, we observed a statistically significant difference (*p*-value  = 0.012, Figure 3C) in the level of centromeric Scm3p in early mitotic cells (300 minute, Figure 3A, 3B) between the control (*SCM3*) and *GALSCM3* strains. We confirmed the latter observations in an additional cell cycle experiment in which *GALSCM3* strain was synchronized in G2/M with nocodazole and released into alpha factor (Figure S6). We did not observe a depletion of Scm3p in *GALSCM3* strains at *CEN* DNA in G2/M cells (0 min, Figure S6). We conclude that the mitotic depletion of Scm3p at *CEN* is affected in *GALSCM3* strains.

### Overexpression of *SCM3* (*GALSCM3*) reduces viability in a subset of kinetochore mutants and leads to premature separation of sister chromatids

**Figure 4:**
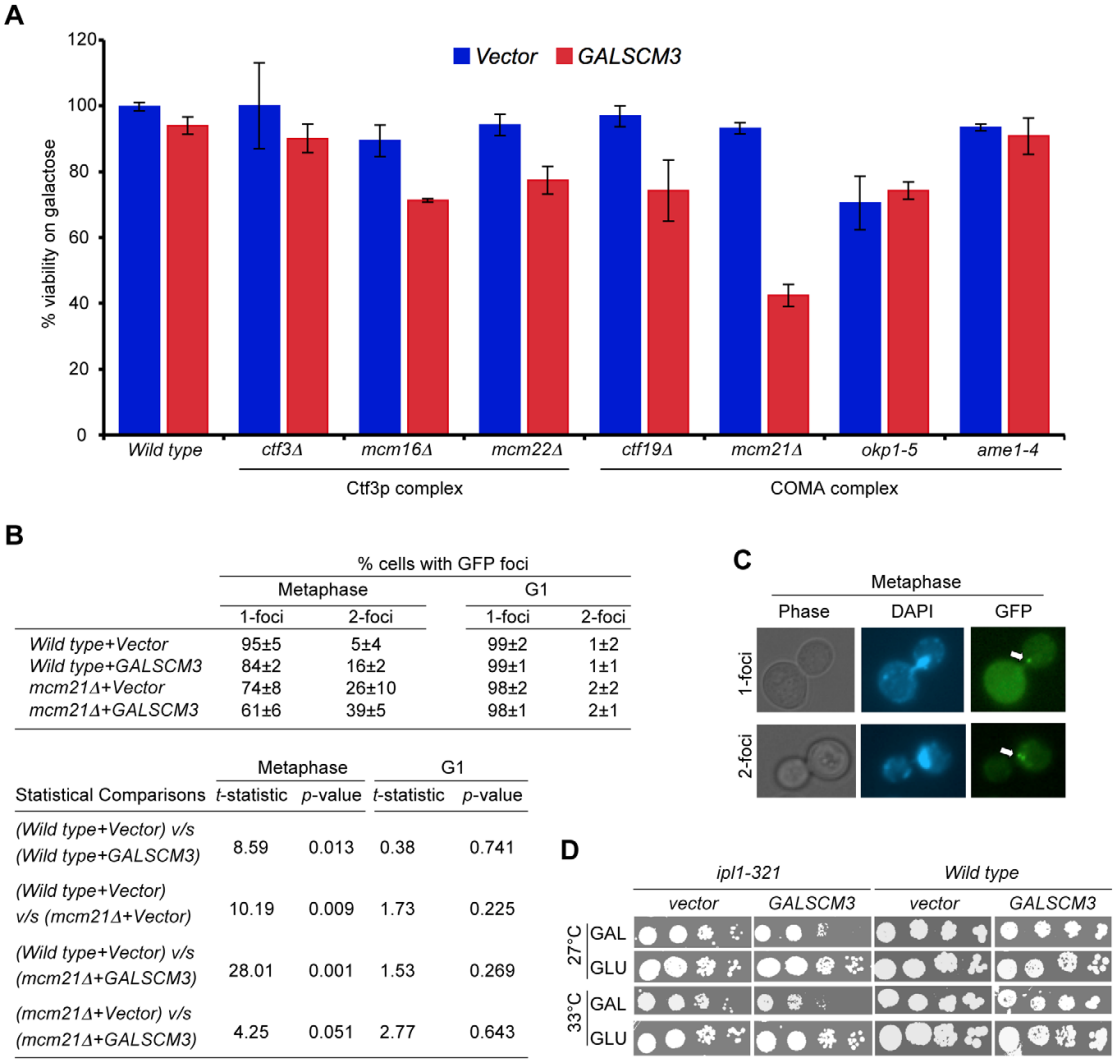
Overexpression of *SCM3* causes reduced viability in a subset of kinetochore mutants and premature separation of sister chromatids. (A) *GALSCM3* causes reduced viability in kinetochore mutants. A wild-type strain (Y5563) and kinetochore mutants *ctf3Δ* (YPH1712), *mcm16Δ* (YPH1714), *mcm22Δ* (YPH1716), *ctf19Δ* (YPH1713), *mcm21Δ* (YPH1715), *okp1-5* (YPH1678), and *ame1-4* (YPH1676) were transformed with *GALSCM3HA* (pMB1306) or vector (pRS426 *GAL1*). Equal number of cells from three independent transformants for each strain were plated on SC-URA with either glucose (2%) or galactose (2%). At least 2500 colonies were counted and % viability is expressed as the ratio of the number of colonies on galactose over the glucose media. (B) Overexpression of *SCM3* leads to premature separation of sister chromatids in metaphase. Sister chromatid separation was monitored in nocodazole-arrested (metaphase) and alpha factor arrested (G1) cells by counting the number of GFP-LacI foci at the marked pericentromere of ChrIV in a wild-type (SBY818) and *mcm21*Δ (SBY1897) strains overexpressing *SCM3* (pMB1306) or a vector (pRS426 *GAL1*). At least 300 cells were analyzed for each strain. Pair-wise comparisons using Student's *t*-test were done to determine the statistical significance between samples. (C) Representative images of metaphase cells showing 1- and 2-GFP LacI foci (see, white arrows) are shown. (D) Overexpression of *SCM3* causes lethality in *ipl1-321* strain. Serial dilutions (5-fold) of *ipl1-321* (SBY630) and wild type (SBY3) strains containing *GALSCM3HA* (pMB1306) or a vector (pRS426 *GAL1*) were plated on SC-URA with glucose (2%) or galactose (2%) plates and grown at 27°C and 33°C for 5 days.

The chromosome loss phenotype of *GALSCM3* strains may be due to compromised kinetochore function and hence, we examined genetic interactions between *GALSCM3* and genes encoding other kinetochore components, specifically subunits of the COMA and Ctf3p complexes. These proteins interact with Cse4p and are important for maintenance of kinetochore integrity [15], [16]. *GALSCM3* showed reduced viability in a subset of the kinetochore mutants, with the most severe phenotype in the *mcm21Δ* strain (Figure 4A). Deletion of *MCM21* is known to cause defects in pericentromeric cohesion, leading to precocious separation of sister chromatids and premature initiation of anaphase [44]. Since *GALSCM3* showed the most severe phenotype in *mcm21Δ* strains, we examined if *GALSCM3* exacerbated the sister chromatid cohesion defect of *mcm21Δ* strains. We monitored the segregation of sister chromatids in metaphase cells by assaying the binding of GFP-LacI to operator sequences inserted 12-kb from the centromere (pericentromere) on Chromosome IV [44]. Wild-type strains arrested in metaphase show a predominance of cells with a single GFP-LacI focus marking two closely associated sister chromatids, whereas premature separation of sister chromatids results in the appearance of two GFP-LacI foci [44]. Consistent with previous findings, we observed a low incidence of two GFP-LacI foci in wild-type cells (5%) compared to that in *mcm21Δ* cells (26%) (Figure 4B, 4C). Strains overexpressing *SCM3* showed a higher incidence of two GFP-LacI foci in wild-type (16%) as well as *mcm21Δ* strains (39%) (Figure 4B, 4C) suggesting that overexpression of *SCM3* enhances premature separation of sister chromatids. The higher incidence of two GFP-LacI foci was observed in mitotic cells but not in G1-arrested cells (Figure 4B). *mcm21Δ* cells depend on the *IPL1* biorientation checkpoint for viability [44]. Similarly, we found that *GALSCM3* leads to growth defects in the *ipl1-321* strain but not in wild-type strains (Figure 4D). Together, these results show that overexpression of *SCM3* enhances premature separation of sister chromatids.

### Scm3p can associate with *CEN* DNA independently of Cse4p

**Figure 5:**
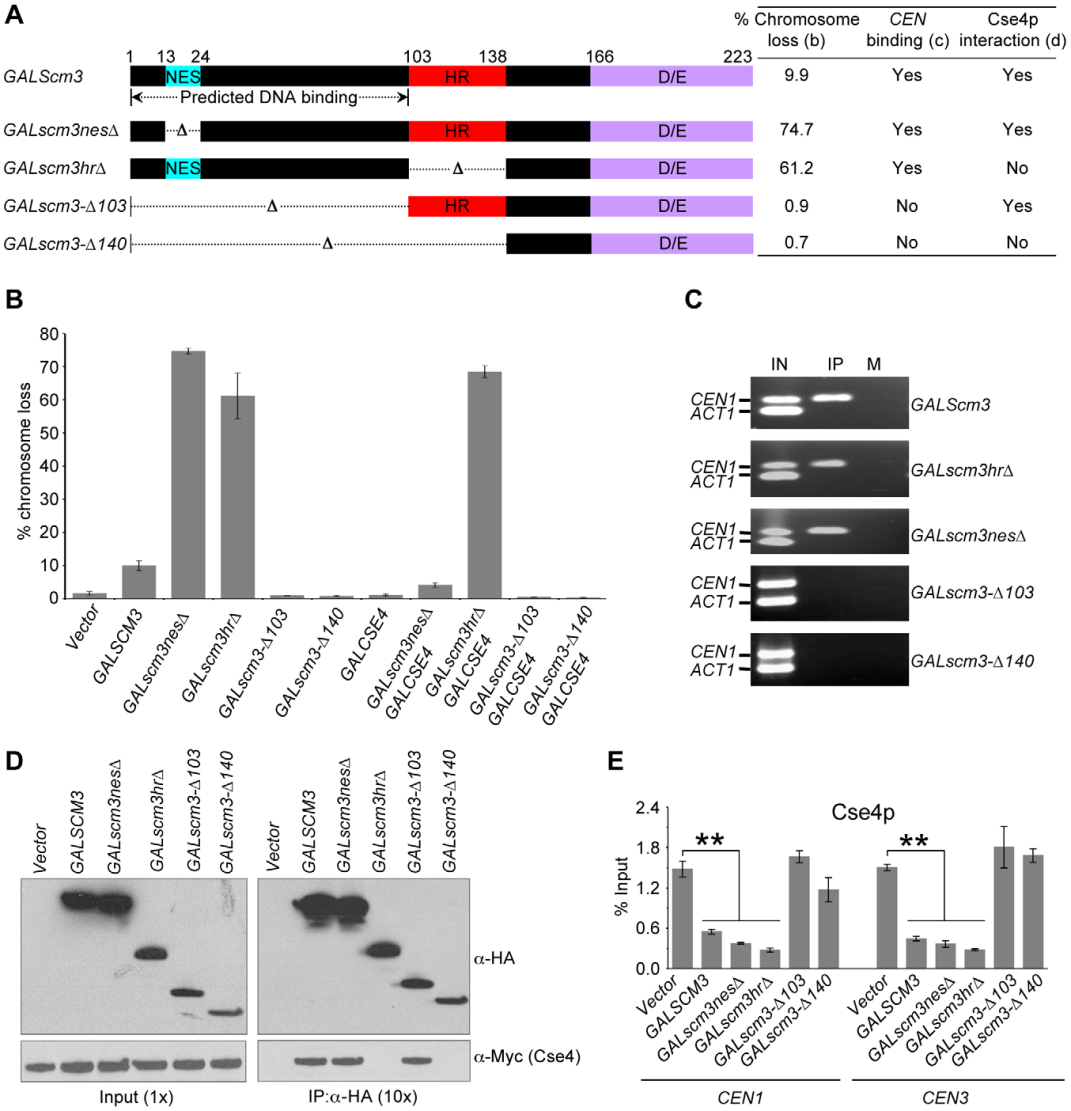
Scm3p can associate with *CEN* DNA independently of Cse4p, and the N-terminus of Scm3p mediates its centromeric association. (A) Schematic of full-length Scm3p and its allelic mutant proteins constructed using gene deletion approach. NES: nuclear export signal; HR: heptad repeat; and D/E: C-terminus acidic domain. Results from chromosome loss (B), *CEN*-binding (C) and Cse4p interactions (D) are summarized in the table. (B) Overexpression of N-terminal mutants (*scm3-Δ103HA*, *scm3-Δ140HA*) does not result in increased chromosome loss. Reporter strains with chromosome fragment (YPH1018) transformed with vector (pRS426 *GAL1*), *GALSCM3HA* (pMB1306), *GALscm3nesΔHA* (pMB1393), *GALscm3hrΔHA* (pMB1455), *GALscm3-Δ103HA* (pMB1520), *GALscm3-Δ140HA* (pMB1521), *GALCSE4* (pMB1147), *GALscm3nesΔHA*, *GALCSE4* (pMB1393, pMB1147), *GALscm3hrΔHA*, *GALCSE4* (pMB1455, pMB1147), *GALscm3-Δ103HA*, *GALCSE4* (pMB1520, pMB976), *GALscm3-Δ140HA*, *GALCSE4* (pMB1521, pMB976) were assayed for chromosome loss as described in Figure 1 (C). (C) N-terminal mutants of Scm3 (*scm3-Δ103HA*, *scm3-Δ140HA*) do not bind *CEN* DNA. ChIP experiments were done using wild type strain (YMB6398 or JG595) with *GALSCM3HA* (pMB1306), *GALscm3nesΔHA* (pMB1393), *GALscm3hrΔHA* (pMB1455), *GALscm3-Δ103HA* (pMB1520), *GALscm3-Δ140HA* (pMB1521) grown in minimal media with galactose (2%) for 12 hours at 30°C and immunoprecipitated with α-HA, and α-GST (mock) antibodies. Enrichment of Scm3p and its allelic mutant forms at *CEN1*, and *ACT1* (internal control) is determined by PCR. Lanes: IN (input), IP (DNA from chromatin immunoprecipitation using α-HA antibodies), and M (DNA from chromatin immunoprecipitation using α-GST antibodies). (D) Western blots of proteins copurifying with Scm3p or its allelic mutant forms. Extracts from cells coexpressing Myc-tagged Cse4p from its native promoter (Cse4-12Myc) and HA-tagged Scm3p or *scm3* mutant proteins expressed from *GAL1* promoter were used in immunoprecipitation (IP) experiments using anti-HA conjugated agarose beads. Eluted proteins were analyzed by western blotting with anti-HA and anti-Myc antibodies. Ten-fold more protein was loaded for IP samples. (E) Overexpression of Scm3p and its mutant alleles (*GALscm3nesΔHA* and *GALscm3hrΔHA*) cause reduction in Cse4p at centromeres. ChIP experiments were done using wild type strain expressing Cse4p-Myc from its endogenous promoter (YMB6398 or JG595) with vector (pRS426 *GAL1*), *GALSCM3HA* (pMB1306), *GALscm3nesΔHA* (pMB1393), *GALscm3hrΔHA* (pMB1455), *GALscm3-Δ103HA* (pMB1520), *GALscm3-Δ140HA* (pMB1521) grown in minimal media with galactose (2%) for 12 hours at 30°C and immunoprecipitated with α-Myc, and α-GST (mock) antibodies. Enrichment of Cse4p to *CEN1* and *CEN3* DNA was determined by qPCR and is shown as % input. Average from at least three independent experiments ± standard error is shown.

Our observation that *GALSCM3* causes reduction in *CEN*-bound Cse4p led us to postulate that the chromosome loss phenotype of *GALSCM3* may be due to centromeric association of Scm3p devoid of Cse4p. Hence, we determined if *CEN*-association of Scm3p requires bound Cse4p. Previous studies of Scm3p have defined two essential domains, a nuclear export signal (NES) that presumably mediates export of Scm3p from the nucleus, and a coiled-coil heptad repeat (HR) domain that mediates interaction with Cse4p [19], [20]. Since deletion of NES and HR domains from endogenous *SCM3* causes lethality [20], we used galactose inducible forms of mutant *scm3* alleles for our analysis. We constructed plasmids expressing *scm3nesΔ* (amino acid residues 13–24 deleted) and *scm3hrΔ* (amino acids 101–139 deleted) from *GAL1* promoter (Figure 5A). Our results showed that *GALscm3nesΔ* and *GALscm3hrΔ* strains exhibit a 6- and 8-fold higher chromosome loss than *GALSCM3* strains (Figure 5B). We reasoned that the chromosome loss phenotype of strains overexpressing *scm3nesΔ*, which can interact with Cse4p, but not *scm3hrΔ*, which lacks the Cse4p interacting domain, would be suppressed by *GALCSE4* [20]. Indeed, *GALCSE4* suppresses the chromosome loss phenotype of *GALscm3nesΔ* strains but not *GALscm3hrΔ* strains (Figure 5B). ChIP results showed that both *GALscm3nesΔ* and *GALscm3hrΔ* can associate with *CEN* DNA (Figure 5C). Co-immunoprecipitation experiments verified previous observations that a deletion of the HR domain of Scm3p but not the NES abolishes interaction with Cse4p (Figure 5D) [20]. Since *GALscm3hrΔ* does not interact with Cse4p and can still bind *CEN* DNA, we conclude that Scm3p can associate with *CEN* DNA independent of its interaction with Cse4p.

### The N-terminus of Scm3p is required for centromeric association of Scm3p

If the *GALSCM3-*induced chromosome loss phenotype is due to centromeric association of Scm3p devoid of Cse4p, overexpression of *scm3* alleles that do not associate with *CEN* DNA should not affect chromosome segregation. To identify the putative DNA interacting domain of Scm3p, we used computational analysis using BindN-RF [45] and MEME [46] software. These analyses suggested that the first 100 amino acids of N-terminus of Scm3p exhibit properties of DNA binding sequences (Figure S7); therefore, we constructed Scm3p variants with a deletion of the putative *CEN* DNA interacting motif (amino acid residues 1–103) either alone or in combination with the HR domain (amino acid residues 1–140) (Figure 5A). Consistent with our hypothesis, *GALscm3-Δ103* or *GALscm3-Δ140* strains do not exhibit a chromosome loss phenotype (Figure 5B) nor do the mutant proteins associate with *CEN* DNA (Figure 5C). As expected, results of co-immunoprecipitation showed that Scm3-Δ103p interacts with Cse4p, while Scm3-Δ140p does not (Figure 5D). Consistent with the lack of chromosome loss phenotype, overexpression of *scm3-Δ103* and *scm3-Δ140* alleles do not affect the level of Cse4p at *CEN* DNA, whereas the overexpressed *scm3nesΔ*, *scm3hrΔ* alleles showed about 3- to 5-fold reduction in the levels of Cse4p at *CEN1* and *CEN3* relative to that of the vector control strain (Figure 5E). Our results show that the N-terminus of Scm3p mediates its association with *CEN* DNA and overexpression of N-terminal *scm3* mutants (*scm3-Δ103* and *scm3-Δ140*) that fail to associate with centromeric chromatin do not result in increased chromosome loss.

### Overexpression of *HJURP* causes chromosome loss in budding yeast and mitotic defects in human cell lines

**Figure 6:**
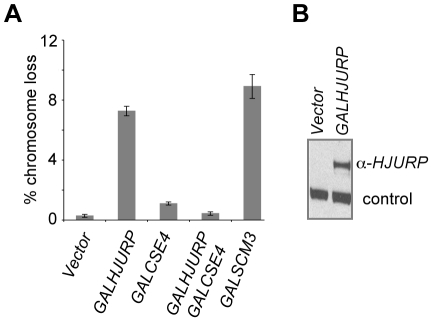
Overexpression of *HJURP* causes chromosome missegregation in budding yeast. (A) Overexpression of *HJURP* leads to chromosome loss in budding yeast. Reporter strains with chromosome fragment (YPH1018) transformed with a vector (pRS426 *GAL1*), *GALHJURP* (pMB1490), *GALCSE4* (pMB1147), *GALHJURP*, *GALCSE4* (pMB1490, pMB1147), and *GALSCM3HA* (pMB1306) were assayed for chromosome loss as described in Figure 1 (C). (B) Western blot analysis showing galactose induced expression of *HJURP*. Whole cell protein extracts prepared from wild type strains (YPH1018) transformed with *GALHJURP* (pMB1490), or vector (pRS426 *GAL1*). Strains were grown to logarithmic phase of growth in synthetic media and gene expression induced in the presence of galactose (2%) at 30°C for 12 hours. Blots were probed with anti-*HJURP*. A non-specific band served as a loading control.

Overexpression of *HJURP* has been observed in mammalian cancer cell lines, such as lung and breast cancers [38], [39]. Based on our results with *GALSCM3*, we tested if overexpression of *HJURP* leads to mitotic defects in yeast. We cloned *HJURP* into a yeast expression vector allowing regulated expression of the gene from a *GAL1* promoter. A wild-type reporter strain overexpressing *HJURP* exhibits a chromosome loss phenotype (7.3±0.3%) similar to that observed for *GALSCM3* (8.9±0.8%) (Figure 6A). *GALCSE4* suppresses the chromosome loss phenotype of the *GALHJURP* strain to the same extent as that observed for strains co-expressing *GALSCM3* and *GALCSE4* (Figure 6A). We confirmed galactose-induced expression of *GALHJURP* by western blotting (Figure 6B).

**Figure 7:**
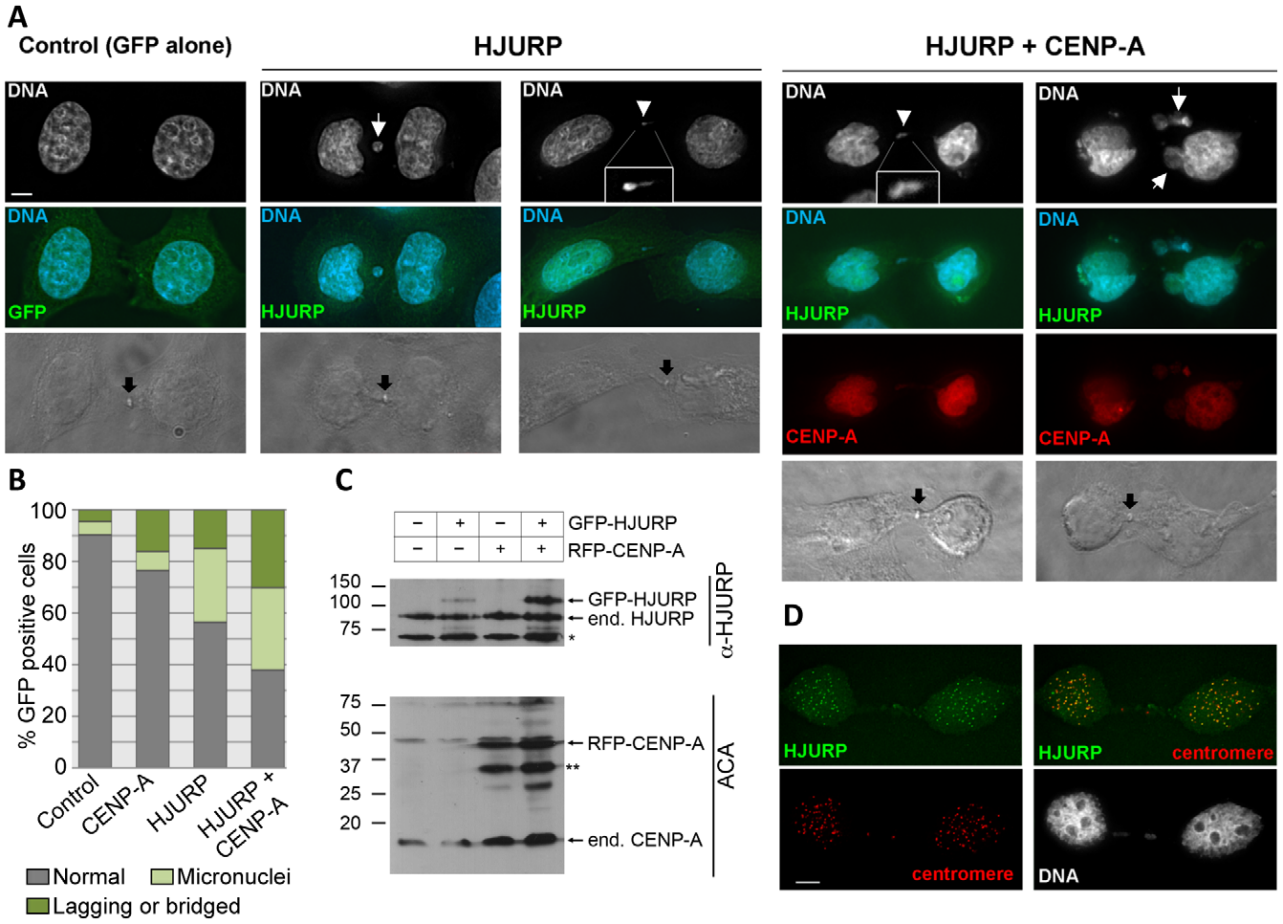
Chromosome segregation errors in human cells over-expressing of *HJURP* and *CENP-A.* (A) HeLa cells were transient transfected with *GFP-HJURP* and/or *RFP-CENP-A*. Cells were blocked in thymidine for 20 hours and released for 12 hours to enrich for cells that have recently undergone mitosis (early G1). Cells in early G1 were identified by the presence of a midbody (black arrow). DNA was stained with DAPI. Micronuclei (white arrow), chromosome bridges or lagging chromosomes (white arrowhead) were evident in cells over-expressing *HJURP*, and both *HJURP* and *CENP-A*. (B) The degree of micronuclei and lagging or bridged chromosomes were quantified in cell transiently over-expressing *RFP-CENP-A*, *GFP-HJURP* or *RFP-CENP-A* and *GFP-HJURP*. While individual over-expression of *CENP-A* or *HJURP* yielded rates of chromosome abnormalities greater than controls, the highest degree of micronuclei and lagging or bridged chromosomes was observed with co-overexpression of both *CENP-A* and *HJURP*. Values are the average from two independent experiments, >100 cells per experiment. (C) Western blot showing expression of *GFP-HJURP* and *RFP-CENP-A* in HeLa cells. Asterisk represents non-specific bands. (D) Cells over-expressing *GFP-HJURP* were pre-extracted in 0.1% Triton prior to fixation. Centromeres were identified using anticentromere autosera. *GFP-HJURP* colocalizes with centromeres demonstrating that over-expressed *HJURP* is able to localize similar endogenous *HJURP*. Scale bar equals 5 micron.

The chromosome loss phenotypes observed upon Scm3p and *HJURP* overexpression in *S. cerevisiae* led us to ask if increased levels of *HJURP* in human cells would also result in chromosome segregation errors. HeLa cells were transfected with a construct to express GFP-tagged *HJURP*. The population was enriched for cells that had recently undergone chromosome segregation by single thymidine block followed by a 12 hr release. The number of micronuclei and lagging or bridged chromosomes (Figure 7A) were assessed as a measure of chromosome missegregation. Ninety percent of control cells (±7%) transfected with GFP vector alone showed no indication of abnormal chromosome segregation; however, when *HJURP* was overexpressed, only 56% (±19%) of nuclei appeared normal. Cells overexpressing *HJURP* had an increased number of recently divided nuclei pairs that contained micronuclei (29±8%) or lagging chromosomes (15±11%) relative to controls (Figure 7B).

Studies with human breast cancer cell lines and primary breast tumors have shown that *HJURP* mRNA levels were significantly correlated with *CENP-A* mRNA levels [39]; hence, we tested whether co-overexpression of *CENP-A* and *HJURP* exacerbated the chromosome missegregation observed with *HJURP* overexpression alone. Cells overexpressing *CENP-A* exhibited an increased incidence of lagging chromosomes (16±3%) compared with controls, not as severe as the effect of *HJURP* overexpression. However, when *CENP-A* and *HJURP* were co-overexpressed in the same cell, the incidence of micronuclei (32±18%) and lagging chromosomes (30±17%) was increased relative to *CENP-A* or *HJURP* overexpression alone (Figure 7B). Increased rates of chromosome loss with overexpression of both *CENP-A* and *HJURP* are consistent with observations in breast cancer where there is a correlation between increased levels of *HJURP* and *CENP-A* [39]. Overexpression of both *CENP-A* and *HJURP* may result in stabilization of *HJURP* (Figure 7C). Alternatively, overexpression of *CENP-A* and *HJURP* may independently lead to a phenotype that is more susceptible to acquiring a tumorigenic potential.

## Discussion

Here we report that misregulation of Scm3p/HJURP causes defects in kinetochore function and results in chromosome instability in yeast and human cells. We used budding yeast to show that overexpression of *SCM3* exhibits reduced viability in kinetochore mutants, premature separation of sister chromatids and lower levels of Cse4p and histone H4 at centromeres. To understand the molecular basis of the chromosome loss phenotype of *GALSCM3* strains, we created strains overexpressing mutant alleles of *SCM3*, and these studies have shown that: a) Scm3p can associate with centromeres independently of its association with Cse4p, b) the N-terminal domain of Scm3p mediates its association with centromeric chromatin, and c) centromeric association of Scm3p devoid of Cse4p is the likely cause of the chromosome loss phenotype of *GALSCM3* strains. Our results establish that balanced stoichiometry of Scm3p and its human homolog *HJURP* are critical for maintenance of genome stability.

Since mutations affecting the HR, NES, and N terminus of Scm3p are lethal, limiting their usefulness, we used overexpression alleles to gain insight into Scm3p function. Overexpression studies have been conducted in other systems to understand the molecular mechanisms of chromosome segregation and kinetochore assembly. For example, overexpression of wild-type or mutant forms of *CENP-A* or its homologs in different organisms such as flies, budding and fission yeasts, leads to chromosome segregation defects [34]–[36]. In flies, excess *CENP-A* promotes the formation of ectopic kinetochores [36]. In budding yeast, excess Cse4p is degraded by ubiquitin mediated proteolysis [41], [47], however a mutant form of Cse4p (Cse4K16R) can be stably overexpressed [48], and such overexpression results in Cse4p mis-localization and chromosome loss [35]. Our results showed that the increased chromosome loss of *GALSCM3* strains does not appear to be due to spreading of Scm3p to non-centromeric regions, as we found no evidence for mis-localization of overexpressed Scm3p to non-*CEN* regions.

*In-vitro* studies in budding yeast have shown that Scm3p directly binds and forms a stoichiometric complex with Cse4p and histone H4 [19]. Similarly, *HJURP* in humans also interacts at a stoichiometric ratio with CENP-A/H4 tetramers [27]. Our results here have shown that overexpression of *SCM3* adversely affects the incorporation of Cse4p/H4 into centromeric chromatin as is evident from the reduction in the levels of Cse4p and H4 at the *CEN* DNA and suppression of *GALSCM3*-induced chromosome loss phenotype by *GALCSE4* or *GALH4* (Figure 1C, 1E, 1F). We propose that *GALSCM3* leads to defects in kinetochore integrity and that this contributes to reduced viability in kinetochore mutants such as *mcm21Δ*. Previous reports have shown that *mcm21* genetically interacts with *cse4* and *ipl1* mutants and *mcm21Δ* strains exhibit precociously separation of sister chromatids in metaphase [44]. It is possible that the lower levels of centromeric Cse4p in *GALSCM3* strains contribute to altered pericentromeric cohesion, which in turn results in premature separation of sister chromatids in the *GALSCM3* strains. Defect in pericentromeric cohesion has been previously reported for *cse4* mutants [49].

**Figure 8:**
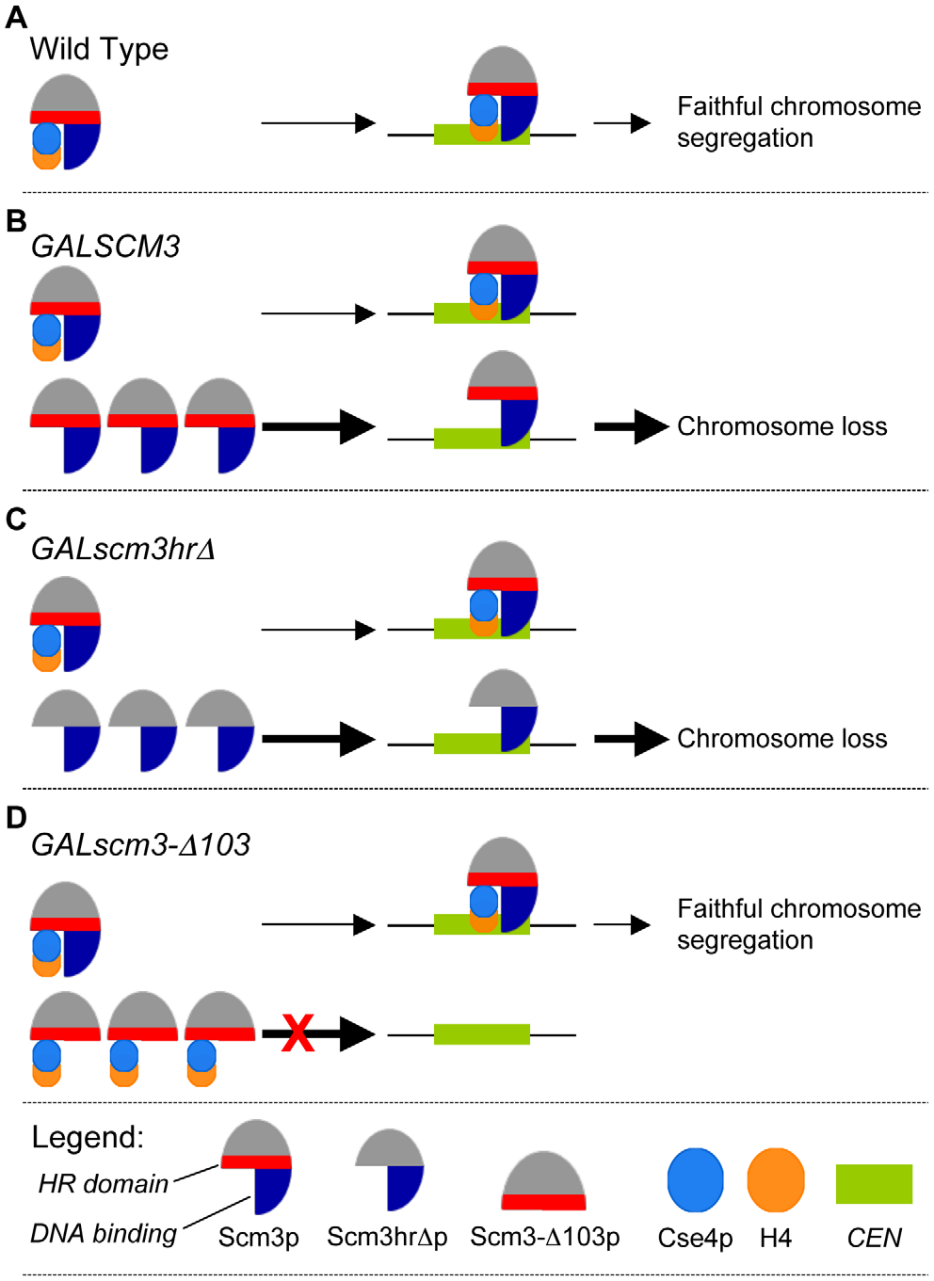
Schematic model for *GALSCM3*-induced chromosome instability in budding yeast. (A) In wild type cells, Scm3p forms a stoichiometric complex with Cse4p/H4 tetramers and mediates the assembly of Cse4p at the *CEN* DNA resulting in faithful chromosome segregation. (B) Centromeric association of Scm3p devoid of Cse4p contributes to the chromosome loss phenotye of *GALSCM3* strains. In *GALSCM3* expressing cells, there is an excess of Scm3p compared to the available pools of Cse4p and H4. Centromeric association of Scm3p devoid of Cse4/H4 leads to chromosome loss. Support for this is based on chromosome loss, ChIP experiments and suppression analysis from Figure 1. (C) Scm3p devoid of Cse4p can associate with centromeric DNA. *GALscm3hrΔ* that cannot interact with Cse4p can bind *CEN* DNA and association of Scm3p devoid of Cse4p leads to the chromosome loss phenotype of *GALscm3hrΔ* strains (Figure 5). (D) The N-terminus of Scm3p defines the centromeric association domain of Scm3p. Overexpression of *scm3* mutants (*GALscm3-Δ103*) that can interact with Cse4p but do not associate with *CEN* DNA do not exhibit increased chromosome loss (Figure 5). Thick arrows represent excess Scm3p or its allelic mutant forms. Taken together our data support the model that centromeric association of Scm3p devoid of Cse4p contributes to the chromosome loss phenotype of *GALSCM3* strains.

We propose a model whereby Scm3p overexpression leads to increased levels of unbound Scm3p, which localizes to *CEN* DNA and interferes with the productive association of Cse4p/H4-Scm3p complexes, resulting in decreased Cse4p incorporation, defects in kinetochore function, and, ultimately, chromosome loss (Figure 8). The model posits that Scm3p devoid of Cse4p can associate with *CEN* DNA, and it correctly predicts that mutant alleles of *scm3* that fail to associate with *CEN* DNA should not affect chromosome segregation (Figure 5B). Mutant alleles of *scm3* deleted of the N terminus (*GALscm3-Δ103)* either alone or in combination with the HR domain (*GALscm3-Δ140*) do not associate with *CEN* DNA (Figure 5C). Our results are consistent with *in vitro* studies demonstrating a DNA-binding domain located within the N-terminal 113 of Scm3p (Carl Wu, personal communication). That *GALscm3hrΔ*, which fails to bind Cse4p (Figure 5D), is still found associated with *CEN* DNA (Figure 5C) indicates that *CEN* DNA association of Scm3p is not dependent on Cse4p interaction even though centromeric localization of Cse4p is dependent on Scm3p [18]–[20], [29]. The higher chromosome loss phenotype of G*ALscm3hrΔ*, and *GALscm3nesΔ* strains compared to that of *GALSCM3* strains suggests that these mutant alleles exhibit a dominant interfering phenotype and this may be due to additional roles of the HR and NES domains. For example, Shivaraju *et al.* have reported that expression of *scm3nesΔ* from its native promoter and simultaneous depletion of Scm3p results in checkpoint activation, and G2/M arrest [29]. Future studies should help us better understand the additional roles of different Scm3p domains.

Our results for the cell cycle regulated enrichment of Scm3p in S phase cells correlates with a similar enrichment of Cse4p in S phase cells and is consistent with the role of Scm3p as a Cse4p assembly factor [18]–[20], [29]–[31]. Studies with budding yeast have shown that replication of *CEN* DNA occurs in early S phase [50], and the centromeres transiently detach from the microtubules in order for the replication machinery to pass through the centromeric region [51]. The S phase co-enrichment of Scm3p and Cse4p could likely reflect the *CEN* DNA replication dependent centromeric assembly of Cse4p. Scm3p homologs in fission yeast also exhibits cell cycle dependent centromeric localization pattern and an enrichment in S phase that correlates with the centromeric assembly of Cnp1 [30], [31]. Our results for depletion of Scm3p in early mitotic cells and enrichment in anaphase cells is similar to findings from fission yeast, wherein Scm3p dissociates from *CEN* in metaphase cells and reappears in anaphase [30], [31]. Despite the difference in centromere structure of fission and budding yeast, the overall cell cycle regulated centromeric localization pattern of Scm3p seems to be evolutionarily conserved in these two systems. However, in humans no *HJURP* signals were detected at centromeres in anaphase and telophase cells [26]. The mechanism and physiological relevance of the mitotic depletion of Scm3p or its homologs has not been investigated in fission yeast or human cells. It is possible that the lack of mitotic depletion of Scm3p in *GALSCM3* strains (300 min, Figure 3B) may contribute to the chromosome loss phenotype. Future studies using mutant alleles of *SCM3*, which fail to show mitotic depletion should give us insights into the role of the cell cycle regulated centromeric association of Scm3p.

We have shown that overexpression of *SCM3* or *HJURP* causes chromosome loss in a wild-type yeast strain, and overexpression of *HJURP* leads to mitotic defects in human cells. Unlike our observations in budding yeast where overexpression of *CSE4* suppresses *GALSCM3* induced chromosome loss, overexpression of *CENP-A* does not suppress, but exacerbates the chromosome loss phenotype of human cells overexpressing *HJURP*. We propose that these differences may be due to the composition of centromeric chromatin, timing of incorporation of CenH3 or additional roles of *HJURP* in humans. For example, in contrast to the point centromeres of budding yeast, human centromeres contain a large number of *CENP-A* nucleosomes that are stably propagated through S phase [52], whereas the single budding yeast Cse4p nucleosome is replaced during S phase [43]. Furthermore, *HJURP* occupies the centromere during a limited time in the cell cycle [26]. Therefore, overexpression of *HJURP* would not be expected to deplete stable *CENP-A* nucleosomes. Recently, *HJURP* mediated deposition of *CENP-A* has been shown to be sufficient to recruit centromeric proteins to an ectopic site within the chromatin [53]. Co-overexpression of *CENP-A* and *HJURP* in human cells may result in the increased incorporation of *CENP-A* at non-centromeric loci. As a result, limiting amounts of critical centromere proteins may be titrated away from endogenous centromeres under these conditions leading to chromosome missegregation. Alternatively, the non-centromeric roles of *HJURP* may contribute to chromosome missegregation [38] and this affect may be increased when *CENP-A* is present because of the increased stability of *HJURP* (Figure 7C) [39]. Unlike *HJURP* overexpression, which causes its mislocalization to different genomic regions [38], overexpression of Scm3p does not result in mis-localization to non-centromeric regions (Figure S2A).

Overall, our results show that proper regulation of *SCM3* and *HJURP* contribute to genome stability. The importance of our results with *SCM3/HJURP* is further reinforced by the fact that *HJURP* overexpression has been observed in human cancer cells and is associated with chromosomal aberrations and aneuploidy [38], [39]. Also, altered dosage and mis-localization of kinetochore proteins, such as *CENP-A*, *CENP-H*, *Aurora-B* and *INCENP* has been observed in various types of tumor cells [37]–[39], [54]. The elucidation of mechanisms underlying regulation of *SCM3/HJURP* in humans may help us identify and develop therapeutic targets for cancer therapy.

## Materials and Methods

### Media, strains, plasmids, and growth conditions

All yeast strains and plasmids used in this study are listed in Table 1 and Table 2, respectively. Yeast growth media, and protocols are as described previously [35]. Plasmid pMB1193 (*2 µm LEU2 GAL1/10-SCM3*) was derived by inserting a PCR-amplified *SCM3* fragment between the *BamHI* and *XhoI* sites of pRS425 *GAL1*. The NES domain of *SCM3* was deleted from pMB1306 using QuickChange Site Directed Mutagenesis Kit (Stratagene) resulting in pMB1393 (*2 µm URA3 GAL1/10-scm3nesΔ-HA*). pMB1455 (*2 µm LEU2 GAL1/10-scm3hrΔ-HA*) was constructed by cloning the PCR-amplified *SCM3hrΔ-HA* fragment from pRB923 between *SpeI* and *XhoI* sites of pRS425 *GAL1*, whereas pMB1490 (*2 µm URA3 GAL1/10-HJURP*) was derived from subcloning the *EcoRI -XhoI* fragment of HJURP (clone id 2820741, Open Biosystem), into pRS426 *GAL1*. Plasmids pMB1520 (*2 µm LEU2 GAL1/10-scm3-Δ103HA*) and pMB1521 (*2 µm LEU2 GAL1/10-scm3-Δ140HA*) were constructed by cloning the PCR-amplified *SCM3-Δ103HA*, and *SCM3-Δ140HA* fragments from pMB1306 between *SpeI* and *XhoI* sites of pRS425 *GAL1*. Strains were grown in YPD (1% yeast extract, 2% Bacto-peptone, 2% glucose) or in synthetic medium containing either 2% glucose or 2% galactose and supplements to allow selection of plasmids being tested.

**Table 1:** *S. cerevisiae* strains used in this study.

Strain	Genotype	Reference
YPH1018	*MATα ura3-52 lys2-801 ade2-101 trp1Δ63 his3Δ200 leu2Δ1 CFIII (CEN3L.YPH278) HIS3 SUP11*	P. Hieter
RC154	*MATa ura3-1 leu2,3-112 his3-1 trp1-1 ade2-1 can1-100 Δbar1 SCM3-3Flag::KAN NDC10-13Myc::TRP1*	[18]
RC100	*MATa ura3-1 leu2,3-112 his3-1 trp1-1 ade2-1 can1-100 Δbar1 CSE4-13myc::URA3 pGAL1-10-3HA-SCM3::KAN*	[18]
JG595	*MATa ura3-1 leu2,3-112 his3-1 trp1-1 ade2-1 can1-100 Δbar1 CSE4-12Myc::URA3 SCM3-3Flag::KAN*	[18]
YMB6094	*MATα ura3-52 lys2-801 ade2-101 trp1Δ63 his3Δ200 leu2Δ1 CFIII (CEN3L.YPH278) HIS3 SUP11 CSE4-13Myc::LEU2*	[35]
YMB6398	*MATa ura3-52 lys2-801 ade2-101 trp1Δ63 his3Δ200 leu2Δ1 CSE4-13Myc::LEU2*	[35]
SBY818	*MATa PDS1-18Myc:LEU2 bar1 ura3-1 leu2-3,112 his3-11:pCUP1-12GFP-12LacI:HIS3 trp1-1:256LacO:TRP1 lys2Δ can1-100 ade2-1*	[44]
SBY1897	*MATa PDS1-18Myc:LEU2 bar1 ura3-1 leu2-3,112 his3-11:pCUP1-12GFP-12LacI:HIS3 trp1-1:256LacO:TRP1 lys2Δ can1-100 ade2-1 mcm21ΔKAN*	[44]
SBY630	*MATa ipl1-321 ura3-1 leu2-3,112 his3-11 trp1-1 can1-100 ade2-1*	[44]
SBY3	*MATa ura3-1 leu2-3,112 his3-11 trp1- Δ1 can1-100 ade2-1 bar1-1*	[58]
Y5563	*MATα his3Δ1 leu2Δ0 ura3Δ0 met15Δ0 lyp1Δ can1Δ::MFA1pr-HIS3*	[59]
YPH1676	*MATa ame1-4:TRP1*	[60]
YPH1678	*MATa okp1-5:TRP1*	[15]
YPH1712	*MATa mfa1Δ::MFa1pr-LEU2 can1Δ::MFA1pr-HIS3 ura3Δ0 leu2Δ0 his3Δ1 lys2Δ0 ctf3Δ::natR*	[16]
YPH1713	*MATa mfa1Δ::MFa1pr-LEU2 can1Δ::MFA1pr-HIS3 ura3Δ0 leu2Δ0 his3Δ1 lys2Δ0 ctf19Δ::natR*	[16]
YPH1714	*MATa mfa1Δ::MFa1pr-LEU2 can1Δ::MFA1pr-HIS3 ura3Δ0 leu2Δ0 his3Δ1 lys2Δ0 mcm16Δ::natR*	[16]
YPH1715	*MATa mfa1Δ::MFa1pr-LEU2 can1Δ::MFA1pr-HIS3 ura3Δ0 leu2Δ0 his3Δ1 lys2Δ0 mcm21Δ::natR*	[16]
YPH1716	*MATa mfa1Δ::MFa1pr-LEU2 can1Δ::MFA1pr-HIS3 ura3Δ0 leu2Δ0 his3Δ1 lys2Δ0 mcm16Δ::natR*	[16]
R421	*MATa trp1-1 ura3-52 leu2::PET56 ade2 SCM3deg::NAT CSE4GFP::TRP1*	[20]

**Table 2:** List of plasmids used in this study.

Plasmid	Description	Reference
pRS424-*GAL1*	*2 µm TRP1 GAL1*	[61]
pRS425-*GAL1*	*2 µm LEU2 GAL1*	[61]
pRS426-*GAL1*	*2 µm URA3 GAL1*	[61]
pMB976	*2 µm TRP1 GAL1/10-CSE4-13Myc*	[35]
pMB1147	*2 µm LEU2 GAL1/10-CSE4-13Myc*	[35]
pMB1158	*2 µm TRP1 GAL1/10-HHF1*	[35]
pMB1159	*2 µm TRP1 GAL1/10-H3*	[35]
pMB1193	*2 µm LEU2 GAL1/10-SCM3*	This study
pMB1306	*2 µm URA3 GAL1/10-SCM3-HA*	[62]
pMB1393	*2 µm URA3 GAL1/10-SCM3nesΔ-HA*	This study
pMB1455	*2 µm LEU2 GAL1/10-SCM3hrΔ-HA*	This study
pMB1490	*2 µm URA3 GAL1/10-HJURP*	This study
pMB1520	*2 µm LEU2 GAL1/10-SCM3-Δ103HA*	This study
pMB1521	*2 µm LEU2 GAL1/10-SCM3-Δ140HA*	This study
pOC52	*URA3 PDS1-HA*	Orna Cohen-Fix
pRB835-2	*TRP1 SCM3-3HA*	Richard Baker
pRB914	*TRP1 SCM3nesΔ*	[20]
pRS314	*TRP1 CEN Vector*	[61]
pRB923	*TRP1 SCM3-hrΔ-HA*	[20]

### Chromosome transmission fidelity (*ctf*) and viability assays

We used an assay developed previously [55] to measure the loss of a non-essential reporter chromosome fragment (CF). Reporter strains were grown to logarithmic phase in synthetic media to maintain the CF and plasmids to be assayed. Cultures were then diluted and plated on synthetic medium to maintain plasmid selection with limiting adenine. The loss of non-essential CF leads to red sectors in an otherwise a white colony. Chromosome loss was measured by counting the percentage of colonies that were at least half red, which represents the loss of reporter chromosome during the first cell division. A minimum of 3000 colonies were assayed from three individual transformants for each strain.

Viability assays for Ctf3p and COMA complex strains containing *GALSCM3HA* (pMB1306) or vector (pRS426 *GAL1*) were carried out by plating equal numbers of cells from three independent transformants for each strain on synthetic media with either glucose (2%) or galactose (2%) and grown at 30°C for 5–7 days. At least 2500 colonies were counted for each strain. Percent viability is expressed as the ratio of the number of colonies obtained on galactose media over the number of colonies obtained on glucose plates and normalized to the value of 100.

### Chromatin immunoprecipitation (ChIP) and quantitative PCR

All ChIP experiments were carried out (three biological replicates) as described [35] with minor modifications. Cultures were cross-linked with formaldehyde (1% final concentration) at room temperature for 15 min and excess formaldehyde was quenched with 125 mM glycine for 5 min. Cell pellets were collected by centrifugation and spheroplasts prepared using Zymolyase 100T, followed by sequential washes with PBS (150 mM NaCl, 10 mM sodium phosphate, pH 7.4), and FA buffer (50 mM Na-Hepes pH 7.6, 1 mM EDTA, 1% Triton X-100, 150 mM NaCl, 0.1% Na-deoxycholate). Spheroplasts were resuspended in FA buffer with protease inhibitors (1 mM PMSF, 1 mM benzamidine, 10 µg/ml leupeptin, 10 µg/ml aprotinin) and sonicated on ice at setting 3, 100% duty cycle, for four 12 sec bursts applied 2 min apart to obtain an average fragment size of 400 bp. The resulting soluble fraction was denoted as input and used for chromatin immunoprecipitation. Protein A magnetic beads (Invitrogen Inc.) coated with antibodies were used in immunoprecipitation performed at 4°C for 12 hours. Antibodies used were anti-FLAG (A2220, Sigma), anti-HA (A2095, Sigma), anti-Myc (sc-789 Santa Cruz Inc), anti-histone H4 (clone 62-141-13, Millipore), anti-*HJURP* [26] or anti-GST (negative control). The immunoprecipitated nucleic acid-protein complexes were washed sequentially at room temperature for 5 min each wash in FA buffer (thrice), FA-HS buffer (50 mM Na-Hepes pH 7.6, 1 mM EDTA, 1% Triton X-100, 500 mM NaCl, 0.1% Na-deoxycholate) (twice), RIPA buffer (10 mM Tris-HCl pH 8.0, 1 mM EDTA, 250 mM LiCl, 0.5% Nonidet P40, 0.5% Na-deoxycholate) (twice), 1 x TE pH 8.0 (twice), and finally resuspended in elution buffer (25 mM Tris-HCl pH 7.6, 10 mM EDTA, 0.5% SDS). Immunoprecipitated DNA was recovered after cross-link reversal at 65°C for 16 hours and RNase A/proteinase K treatment, purified by phenol/chloroform/isoamyl alcohol (25∶24∶1) extractions, and ethanol precipitated (−20°C for 12 hours). Enriched DNA was recovered by centrifugation and dissolved in TE (10 mM Tris, 1 mM EDTA, pH 8.0).

Quantitative real time ChIP-PCR was performed using Fast SYBR Green Master Mix in 7500 Fast real Time PCR System (Applied Biosystem Inc.) following manufacturer's instructions. PCR conditions were: denaturation at 95°C for 20 sec, followed by 40 cycles of 95°C for 3 sec, 60°C for 30 sec. The amplification readings were recorded and threshold cycle (Ct) was determined by 7500 Fast System Software v1.4.0 (Applied Biosystem Inc.). The enrichment values were calculated using the ΔΔCt method [56] and are presented as % input.

ChIP-PCR was done using Hotstar-Taq polymerase Master Mix (Qiagen Inc.), with initial denaturation at 94°C for 15 min, followed by 25 cycles of 94°C for 1 min annealing at 54°C for 1 min and extension at 68°C for 10 sec. The linear amplification conditions were determined by various cycles of amplification using serial dilutions of the input DNA. PCR products were separated on 2% agarose gel, stained with ethidium bromide and photographed. The intensity of PCR amplicons was determined using the software ImageJ 1.43u (http://rsb.info.nih.gov/ij). Sequences of primers used in this study are listed in Table 3.

**Table 3:** List of primers used in this study.

Locus	Forward primer (5′ to 3′)	Reverse primer (5′ to 3′)
*CEN1*	CTCGATTTGCATAAGTGTGCC	GTGCTTAAGAGTTCTGTACCAC
*CEN3*	GATCAGCGCCAAACAATATGG	AACTTCCACCAGTAAACGTTTC
*CEN5*	AAGAACTATGAATCTGTAAATGACTGATTCAAT	CTTGCACTAAACAAGACTTTATACTACGTTTAG
*ACT1*	ACAACGAATTGAGAGTTGCCCCAG	AATGGCGTGAGGTAGAGAGAAACC
*IR1*	TACTGGCAAGCACGGAAGGC	AATCCCACGTGCACCCATAC
*IR2*	ATGAATAAGCAAGATCTCAT	CTCGCCTTAACCACTCGGCC
*IR3*	TGGGTCTAAGAGTATGTACGGATGT	GTAGTCTGTTTATCCGTCATATTCCC
*IR4*	GTTCACCAGGTAATAATCACTGACTG	TTGTTGTTATTGGTATTATTAGCAGG
*AGP1*	CCATGAAAGTCCAAGGGAGA	CAATCCTTGTGCCAGACCTT
*CWH43*	AAAAGGAAAAACCCGTTGCT	GAAGGCTTCAGAAACGAACG
*YGL036W*	GGGGGCCACAATAAAGTTAAAAAC	CAAGAACTGGAAACATTACCACCC

### Immunoprecipitation and Western blot analysis

Immunoprecipitation experiments were performed as described previously [18]. Protein samples for western blot analysis were prepared using the TCA procedure as described previously [57], and protein concentration was measured using the Bio-Rad DC protein assay (Bio-Rad, CA). Equal amounts of protein for each sample were size separated on 4–12% gradient polyacrylamide gels and transferred to nitrocellulose membrane. Primary antibodies used were anti-HA (clone 12CA5, Roche), anti-Myc (Z-5, sc-789, Santa Cruz Inc), anti-histone H3 (ab1791-100, Abcam), anti-histone H4 (clone 62-141-13, Millipore), anti-Flag (A-6457, Molecular Probes) or anti-HJURP [26]. Secondary antibodies used were HRP-conjugated sheep anti-mouse IgG (NA931V–Amersham) and HRP-conjugated sheep anti-rabbit IgG (NA934V–Amersham). Rabbit polyclonal antibodies against Tub2p were custom made by Covance, Inc.

### Cell cycle arrest and release experiments

For determining the *CEN* enrichment of Scm3p through the cell cycle, wild-type strain (RC154) with FLAG-tagged Scm3p expressed from its endogenous promoter was used. A wild type strain (JG595) with Myc-tagged Cse4p expressed from its endogenous promoter was used to determine the *CEN* enrichment of Cse4p through the cell cycle. Cells were grown in YPD to logarithmic (LOG) phase at 30°C, treated with 3 µM α-factor (T-6901, Sigma, St. Louis) for 90 minutes to arrest cells in G1, washed, and released into pheromone-free YPD medium. Samples were taken at 15 min time points after release from G1 arrest and used for FACS, protein and ChIP analysis. Additional synchronizations were carried out to confirm the results: cells were arrested in G1 with α-factor as described above and released into YPD medium containing 15 µg/ml nocodazole (M1404, Sigma); cells were arrested in G2/M with nocodazole for 2 hours and released into YPD medium containing 3 µM α-factor (T-6901, Sigma).

To examine the *CEN* enrichment of overexpressed Scm3p (*GALSCM3*) through the cell cycle, strain RC100 with HA tagged Scm3p integrated at its endogenous locus (only copy in the genome) and expressed from *GAL1* promoter was used. Cells were grown in SC media with 2% galactose to logarithmic (LOG) phase at 30°C, treated with 3 µM α-factor (T-6901, Sigma, St. Louis) for 90 minutes for G1 arrest, washed, and released into pheromone-free SC media with 2% galactose medium. Samples were taken at different time points after release from G1 arrest and were used in FACS and ChIP analysis.

### FACS and nuclear morphology analyses

FACS assays was performed to confirm the cell cycle arrest and release. Cells were fixed in 70% ethanol, washed in 0.2 M Tris buffer, treated with RNase A, Proteinase K, stained with propidium iodide, and analyzed using a Becton-Dickinson FACSort flow cytometer and Cell Quest software (BD Biosciences, Boston, MA). Cell cycle stages were determined by examining cell morphology and nuclear position in propidium iodide-stained cells under the Zeiss Axioskop 2 microscope (Carl Zeiss Inc., Thornwood, USA) as described previously [42]. Cell cycle stages were defined as follows: G1, single cells with undivided nuclei; S-phase, cells showing a small bud with undivided nuclei; metaphase, large budded cells with nucleus at the neck; anaphase, large budded cells with elongated/separated nuclei; and telophase, large budded cells with nuclei separated between mother and daughter cells (see Figure 2).

### Human cell culture

HeLa cells were plated 24 hours prior to transfection to poly-lysine coated 12mm square coverslips in a 6-well plate. Cells were incubated with DNA and Effectene transfection reagent (Qiagen) according the manufacturer instructions in Optimem (Invitrogen) for 8 hours and at which time the media was replaced with normal growth media (DMEM, 10% FBS, supplemented with Penicillin and Streptomycin). Beginning 24 hours after transfection, cells were incubated with 2mM thymidine for 20 hours, then released from thymidine by washing cells in PBS and incubating cells in normal growth media supplemented with 20mM deoxycytidine for 12 hours prior to fixation. Cells were fixed after in 4% formaldehyde and blocked in PBS with 2% FBS, 2% BSA and 0.1% TritonX-100. Antibody incubates were conducted for 1 hour at room temperature in blocking buffer. Centromeres were detected using anti-centromere autosera (Antibodies incorporated) and Cy5 conjugated goat-anti-human secondary antibodies (Jackson Labs). DNA was stained using 2 µg/ml 4′,6-diamidino-2-phenylindole (DAPI) and cells were mounted in Prolong Antifade (Invitrogen). Z-stacked images were collected on a Deltavision microscope (Applied Precision Instruments) using a 60X objective. Images were deconvolved and are presented as maximum projections.

## Supporting Information

Figure S1 Overexpression of *SCM3* causes chromosome instability in wild-type strains. Quantification of chromosome loss by half-sector analysis. Reporter strains with chromosome fragment (YPH1018) transformed with vector (pRS426 *GAL1*), *GALSCM3HA* (pMB1306), *GALSCM3* (pMB1193), or *GALSCM3HA* (pMB1306) integrated at *URA3* locus in the genome were plated on SC-URA with limiting adenine and galactose (2%). At least 3000 colonies were counted. Values represent the average and standard error of chromosome loss for three independent transformants and were normalized to the value of 100.
(TIF)

Figure S2 Scm3p and Cse4p are not mislocalized to non-centromeric DNA regions in strains overexpressing *SCM3*. (A) A wild-type strain (RC154) with FLAG-tagged Scm3p expressed from its own endogenous promoter was transformed with vector (pRS426 *GAL1*), or *GALSCM3HA* (pMB1306) and grown in minimal media with galactose (2%) for 12 hours at 30°C. Chromatin immunoprecipitation were done with α-HA (Scm3p expressed from *GAL1* promoter), α-FLAG (Scm3p expressed from its endogenous promoter), and α-GST (mock) antibodies. Enrichment of Scm3p in these strains was assayed by qPCR using primers representing *CEN3*, intergenic (*IR1*, *IR2*, *IR3*, *IR4*), and transcribed (*AGP1*, *CWH43*, *ACT1*, and *YGL036W*) regions. Chromosomal coordinates of these DNA regions were derived from yeast genome database (www.yeastgenome.org) and are as follows: *CEN3* (Chromosome III, 114385-114501), *IR1* (Chromosome III, 163368-163699), *IR2* (Chromosome III, 227628-227969), *IR3* (Chromosome VI, 224045-224329), *IR4* (Chromosome XVI, 520851-521150), *AGP1* (Chromosome III, 76166-76400), *CWH43* (Chromosome III, 146651-146886), *ACT1* (Chromosome VI, 54093-53886), and *YGL036W* (Chromosome VII, 431253-431610). Average from at least three independent experiments ± standard error is shown as % input. *p value <0.05, **p value <0.01, Student's t test. (B) *GALSCM3* strains show reduced levels of *CEN*-associated Cse4p. ChIP experiments were done using wild type strain expressing Cse4p-Myc from its endogenous promoter (YMB6094) with vector (pRS426 *GAL1*), or *GALSCM3HA* (pMB1306) grown in minimal media with galactose (2%) for 12 hours at 30°C and immunoprecipitation were done with α-Myc, and α-GST (mock) antibodies. Enrichment of Cse4p was determined using primers for DNA regions described in (A) above.
(TIF)

Figure S3 Centromeric enrichment of Scm3p is cell cycle regulated. A wild-type strain (RC154) with FLAG-tagged Scm3p expressed from its own endogenous promoter was used. Cells were grown in YPD to logarithmic (LOG) phase, synchronized with α-factor in G1, and released into YPD containing nocodazole (A), or synchronized with nocodazole in G2/M, and released into YPD containing α-factor (B). Samples were taken at time points (min) after release. (FACS): DNA content was determined by FACS analysis. (cell morphology): cell cycle stages were determined based on cell shape and nuclear position by microscopic examination of at least 200 cells for each time point. Numbers represent the percentage of cells in each of the categories (G1, S, M, A, T) as described in Materials and Methods. (ChIP): enrichment of Scm3p at *CEN* DNA was examined by chromatin immunoprecipitation using α-FLAG (Scm3p), and α-GST (mock) antibodies. The immunoprecipitated DNA fragments were purified and used as templates for traditional PCR using primers specific for *CEN1* DNA. To determine the enrichment of Scm3p to *CEN1* DNA, signals obtained from the immunoprecipitated DNA were divided by signals of the corresponding input DNA and normalized to the values from *ACT1* (bottom DNA band in the gel images). The average enrichment from at least three independent experiments, with standard errors is shown. *p value <0.05, **p value <0.01, Student's t test. Lanes: IN (input), IP (chromatin immunoprecipitated DNA with α-FLAG antibodies), and M (chromatin immunoprecipitated DNA with α-GST antibodies).
(TIF)

Figure S4 Expression of Scm3p is not cell cycle regulated. Western blot analysis was done on whole cell protein extracts prepared using samples used for Figure 2, which were taken at time points after release from G1. Western blots were probed with α-FLAG (Scm3p), α-HA (Pds1p), and α-Tub2p (loading control) antibodies.
(TIF)

Figure S5 Centromeric association of Cse4p through the cell cycle. A wild-type strain (JG595) with Myc tagged Cse4p expressed from its own endogenous promoter was grown in YPD, synchronized in G1 with α-factor, washed, and released into pheromone-free YPD medium. Samples were taken at time points (min) after release from G1. (A) DNA content was determined by FACS. (B) Cell cycle stages were determined based on cell morphology and nuclear position by microscopic examination of 200 cells for each time point. (C) Enrichment levels of Cse4p at *CEN1*. ChIP experiments were done using α-Myc (Cse4p), and α-GST (mock) antibodies. The immunoprecipitated DNA fragments were purified and used as templates for traditional PCR using primers specific for *CEN1* DNA. To determine the enrichment of Cse4p to *CEN1* DNA, signals obtained from the immunoprecipitated DNA were divided by signals of the corresponding input DNA and normalized to the values from a background control region, *ACT1* (bottom DNA band in the gel images). The average enrichment from at least three independent experiments, with standard errors is shown. *p value <0.05, **p value <0.01, derived using Student's t test. Lanes: IN (input), IP (chromatin immunoprecipitated DNA with α-Myc antibodies), and M (chromatin immunoprecipitated DNA with α-GST antibodies).
(TIF)

Figure S6 Centromeric enrichment pattern of Scm3p expressed from a *GAL1* promoter (*GALSCM3*) through the cell cycle. Wild-type strain expressing HA tagged Scm3p from *GAL1* promoter (RC100) was grown in minimal media with galactose (2%) at 30°C, treated with nocodazole for 2 hours to synchronize cells in G2/M, washed, and released into minimal media containing α-factor. Samples were taken at time points (min) after release from G2/M arrest. (A) DNA content was determined by FACS. (B) Cell cycle stages were determined based on cell morphology and nuclear position by microscopic examination of 200 cells for each time point. (C) Enrichment levels of Scm3p at *CEN3*. ChIP experiments were done using α-HA (Scm3p), and α-GST (mock) antibodies. The enrichment of Scm3p at *CEN3* was determined by qPCR and is shown as % input. Average from at least three independent experiments ± standard error is shown. No significant differences in enrichment were observed among time points.
(TIF)

Figure S7 Identification of DNA binding sequences of Scm3p. Predicted DNA binding sequences identified by computational analysis using BindN-RF and MEME software are shown in red and marked with an arrow (amino acids 1-103). Amino acids residues predicted to interact with DNA with high affinity are shown as brown color letters. Symbols, NES  =  nuclear export signal; HR  =  Cse4p interacting heptad repeat domain; acidic D/E region, and BR1 and BR2 are basic regions 1 and 2, respectively.
(TIF)
