# Supplementary figures and images for: Misregulation of Scm3p/HJURP Causes Chromosome Instability in Saccharomyces cerevisiae and Human Cells
Source: PLoS Genet. 2011 Sep 29;7(9):e1002303. doi: 10.1371/journal.pgen.1002303 (PMC3183075; doi:10.1371/journal.pgen.1002303)

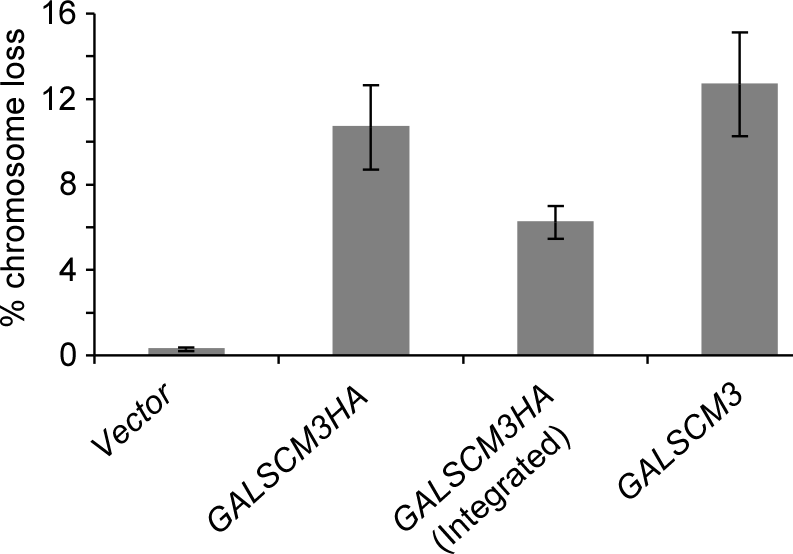

Supplement: Figure S1 — Overexpression of SCM3 causes chromosome instability in wild-type strains. Quantification of chromosome loss by half-sector analysis. Reporter strains with chromosome fragment (YPH1018) transformed with vector (pRS426 GAL1), GALSCM3HA (pMB1306), GALSCM3 (pMB1193), or GALSCM3HA (pMB1306) integrated at URA3 locus in the genome were plated on SC-URA with limiting adenine and galactose (2%). At least 3000 colonies were counted. Values represent the average and standard error of chromosome loss for three independent transformants and were normalized to the value of 100. (TIF) [file pgen.1002303.s001.tif]

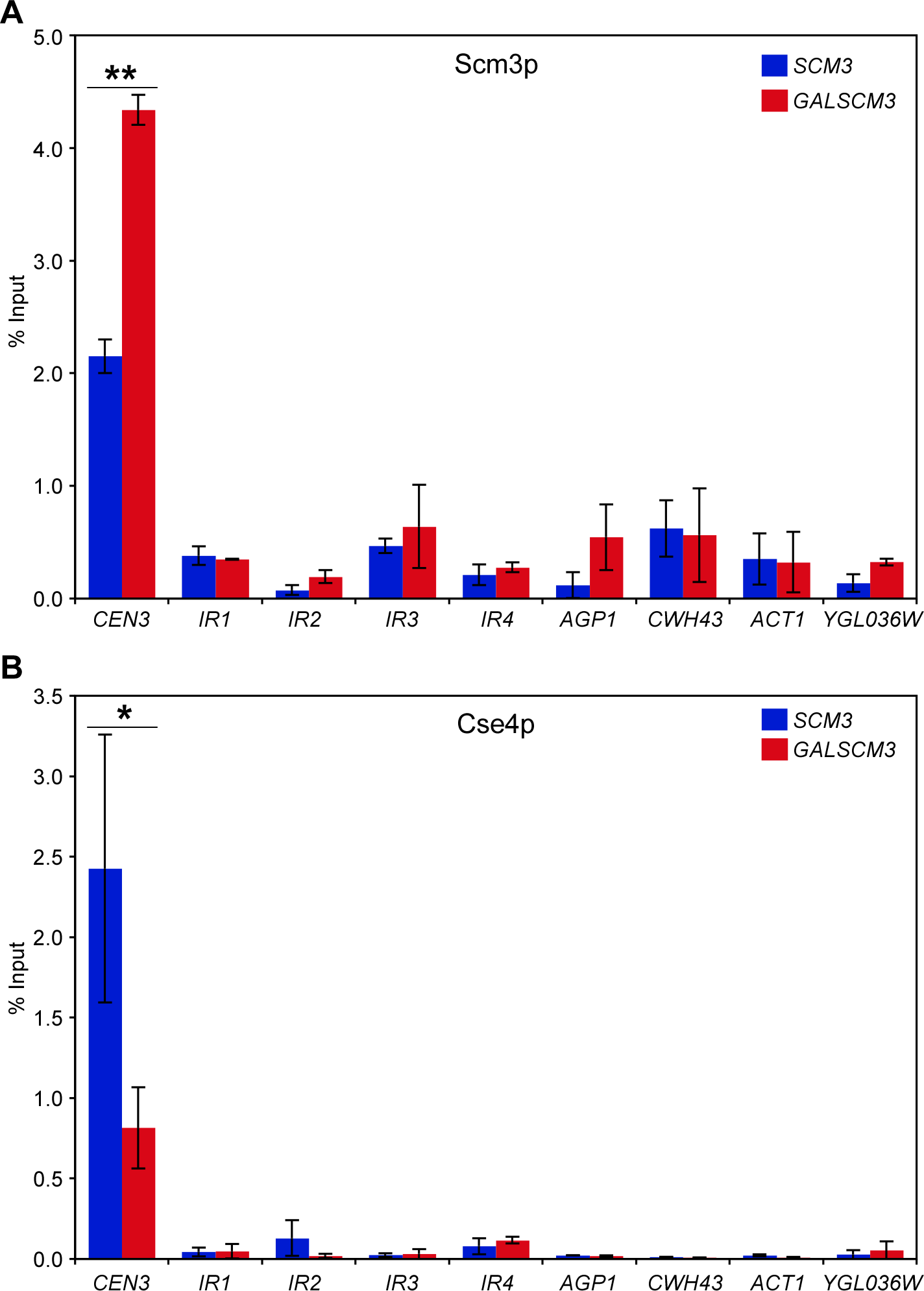

Supplement: Figure S2 — Scm3p and Cse4p are not mislocalized to non-centromeric DNA regions in strains overexpressing SCM3. (A) A wild-type strain (RC154) with FLAG-tagged Scm3p expressed from its own endogenous promoter was transformed with vector (pRS426 GAL1), or GALSCM3HA (pMB1306) and grown in minimal media with galactose (2%) for 12 hours at 30°C. Chromatin immunoprecipitation were done with α-HA (Scm3p expressed from GAL1 promoter), α-FLAG (Scm3p expressed from its endogenous promoter), and α-GST (mock) antibodies. Enrichment of Scm3p in these strains was assayed by qPCR using primers representing CEN3, intergenic (IR1, IR2, IR3, IR4), and transcribed (AGP1, CWH43, ACT1, and YGL036W) regions. Chromosomal coordinates of these DNA regions were derived from yeast genome database (www.yeastgenome.org) and are as follows: CEN3 (Chromosome III, 114385-114501), IR1 (Chromosome III, 163368-163699), IR2 (Chromosome III, 227628-227969), IR3 (Chromosome VI, 224045-224329), IR4 (Chromosome XVI, 520851-521150), AGP1 (Chromosome III, 76166-76400), CWH43 (Chromosome III, 146651-146886), ACT1 (Chromosome VI, 54093-53886), and YGL036W (Chromosome VII, 431253-431610). Average from at least three independent experiments ± standard error is shown as % input. *p value <0.05, **p value <0.01, Student's t test. (B) GALSCM3 strains show reduced levels of CEN-associated Cse4p. ChIP experiments were done using wild type strain expressing Cse4p-Myc from its endogenous promoter (YMB6094) with vector (pRS426 GAL1), or GALSCM3HA (pMB1306) grown in minimal media with galactose (2%) for 12 hours at 30°C and immunoprecipitation were done with α-Myc, and α-GST (mock) antibodies. Enrichment of Cse4p was determined using primers for DNA regions described in (A) above. (TIF) [file pgen.1002303.s002.tif]

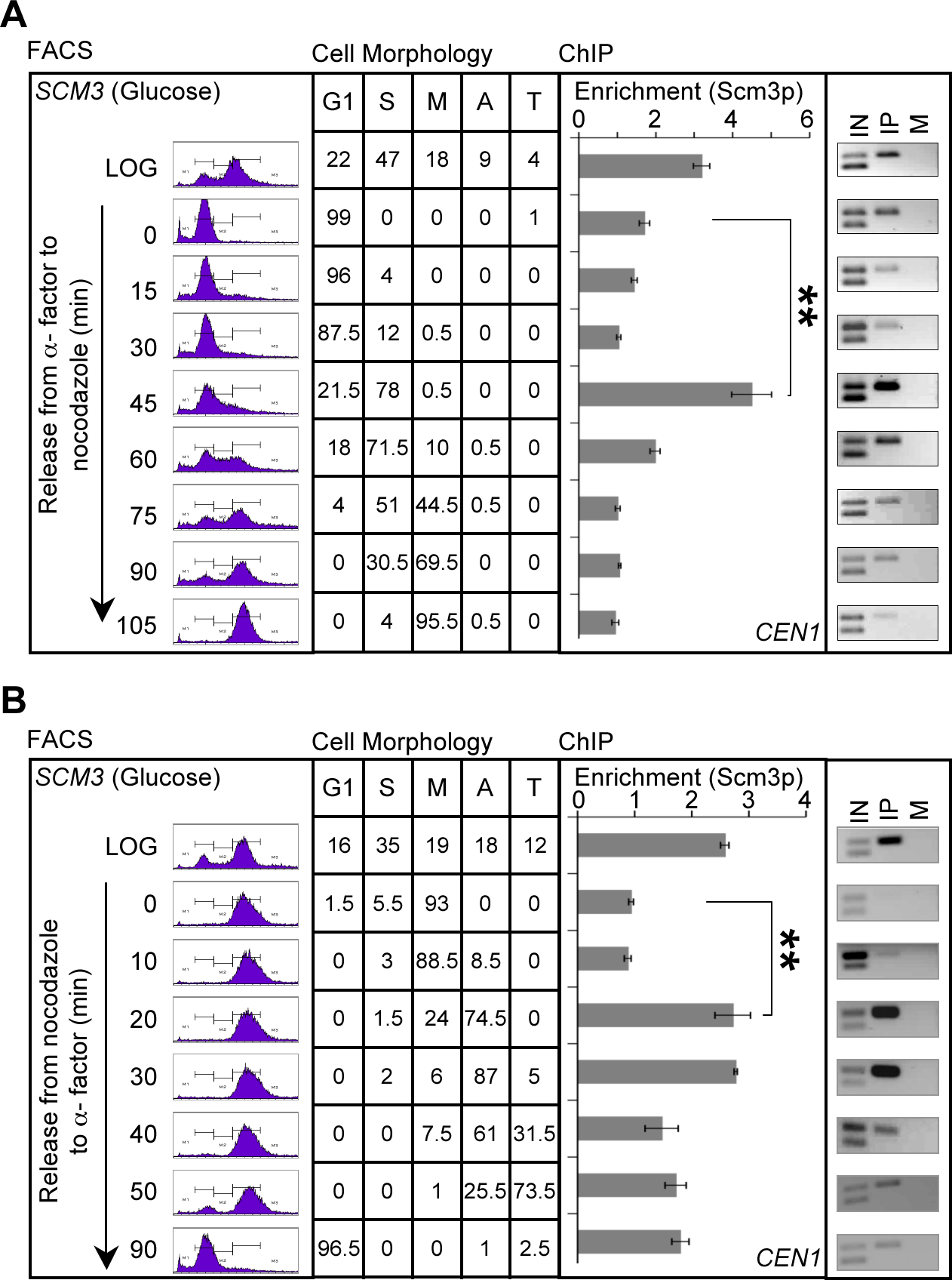

Supplement: Figure S3 — Centromeric enrichment of Scm3p is cell cycle regulated. A wild-type strain (RC154) with FLAG-tagged Scm3p expressed from its own endogenous promoter was used. Cells were grown in YPD to logarithmic (LOG) phase, synchronized with α-factor in G1, and released into YPD containing nocodazole (A), or synchronized with nocodazole in G2/M, and released into YPD containing α-factor (B). Samples were taken at time points (min) after release. (FACS): DNA content was determined by FACS analysis. (cell morphology): cell cycle stages were determined based on cell shape and nuclear position by microscopic examination of at least 200 cells for each time point. Numbers represent the percentage of cells in each of the categories (G1, S, M, A, T) as described in Materials and Methods. (ChIP): enrichment of Scm3p at CEN DNA was examined by chromatin immunoprecipitation using α-FLAG (Scm3p), and α-GST (mock) antibodies. The immunoprecipitated DNA fragments were purified and used as templates for traditional PCR using primers specific for CEN1 DNA. To determine the enrichment of Scm3p to CEN1 DNA, signals obtained from the immunoprecipitated DNA were divided by signals of the corresponding input DNA and normalized to the values from ACT1 (bottom DNA band in the gel images). The average enrichment from at least three independent experiments, with standard errors is shown. *p value <0.05, **p value <0.01, Student's t test. Lanes: IN (input), IP (chromatin immunoprecipitated DNA with α-FLAG antibodies), and M (chromatin immunoprecipitated DNA with α-GST antibodies). (TIF) [file pgen.1002303.s003.tif]

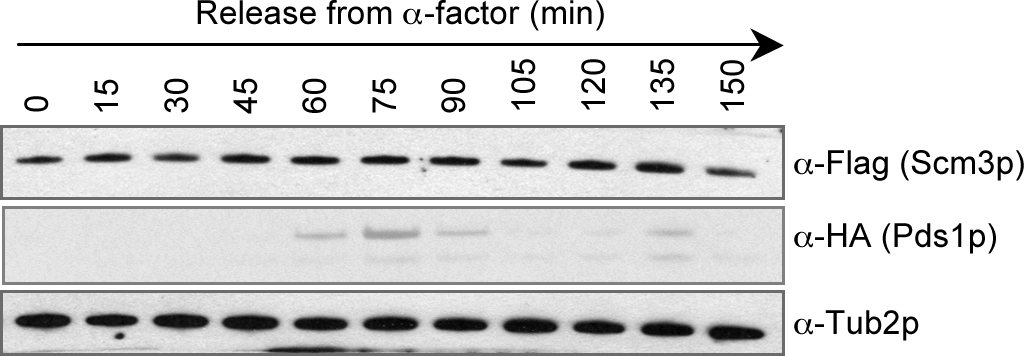

Supplement: Figure S4 — Expression of Scm3p is not cell cycle regulated. Western blot analysis was done on whole cell protein extracts prepared using samples used for Figure 2, which were taken at time points after release from G1. Western blots were probed with α-FLAG (Scm3p), α-HA (Pds1p), and α-Tub2p (loading control) antibodies. (TIF) [file pgen.1002303.s004.tif]

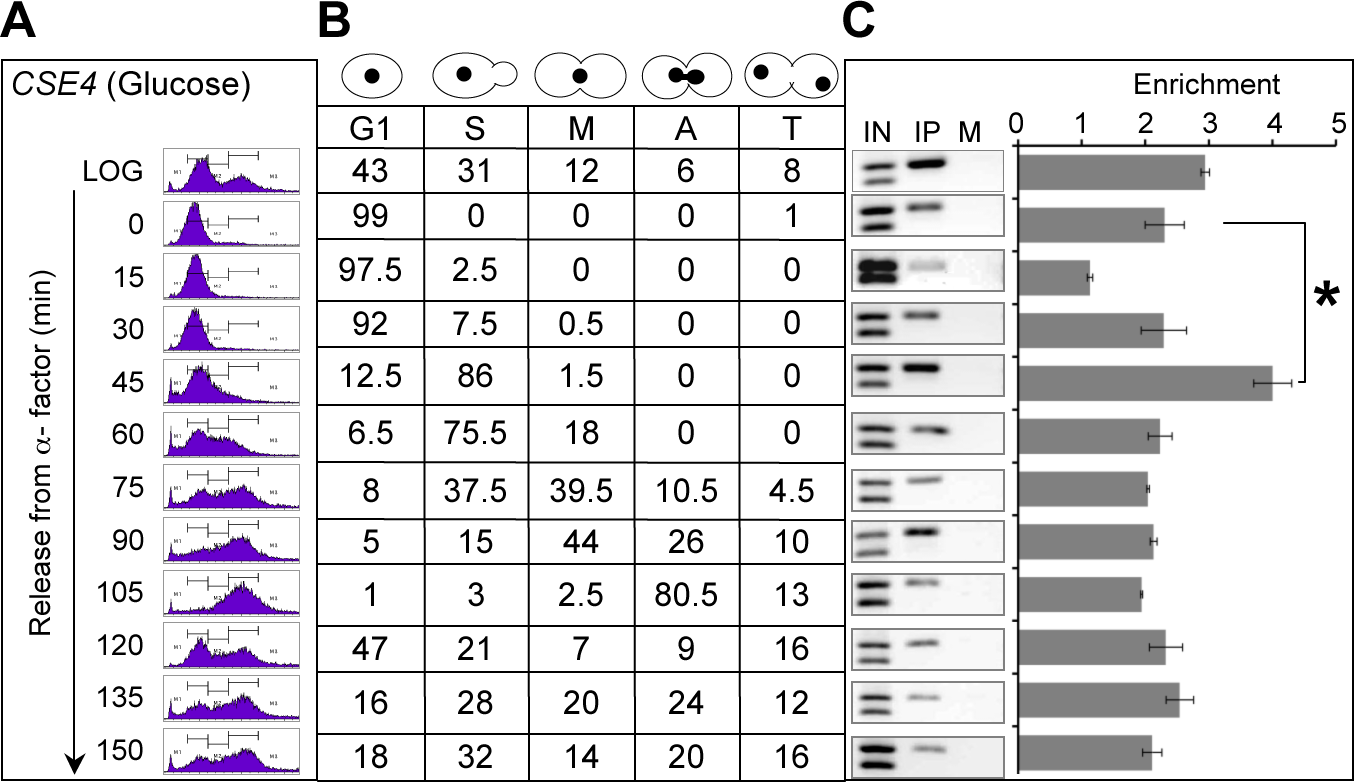

Supplement: Figure S5 — Centromeric association of Cse4p through the cell cycle. A wild-type strain (JG595) with Myc tagged Cse4p expressed from its own endogenous promoter was grown in YPD, synchronized in G1 with α-factor, washed, and released into pheromone-free YPD medium. Samples were taken at time points (min) after release from G1. (A) DNA content was determined by FACS. (B) Cell cycle stages were determined based on cell morphology and nuclear position by microscopic examination of 200 cells for each time point. (C) Enrichment levels of Cse4p at CEN1. ChIP experiments were done using α-Myc (Cse4p), and α-GST (mock) antibodies. The immunoprecipitated DNA fragments were purified and used as templates for traditional PCR using primers specific for CEN1 DNA. To determine the enrichment of Cse4p to CEN1 DNA, signals obtained from the immunoprecipitated DNA were divided by signals of the corresponding input DNA and normalized to the values from a background control region, ACT1 (bottom DNA band in the gel images). The average enrichment from at least three independent experiments, with standard errors is shown. *p value <0.05, **p value <0.01, derived using Student's t test. Lanes: IN (input), IP (chromatin immunoprecipitated DNA with α-Myc antibodies), and M (chromatin immunoprecipitated DNA with α-GST antibodies). (TIF) [file pgen.1002303.s005.tif]

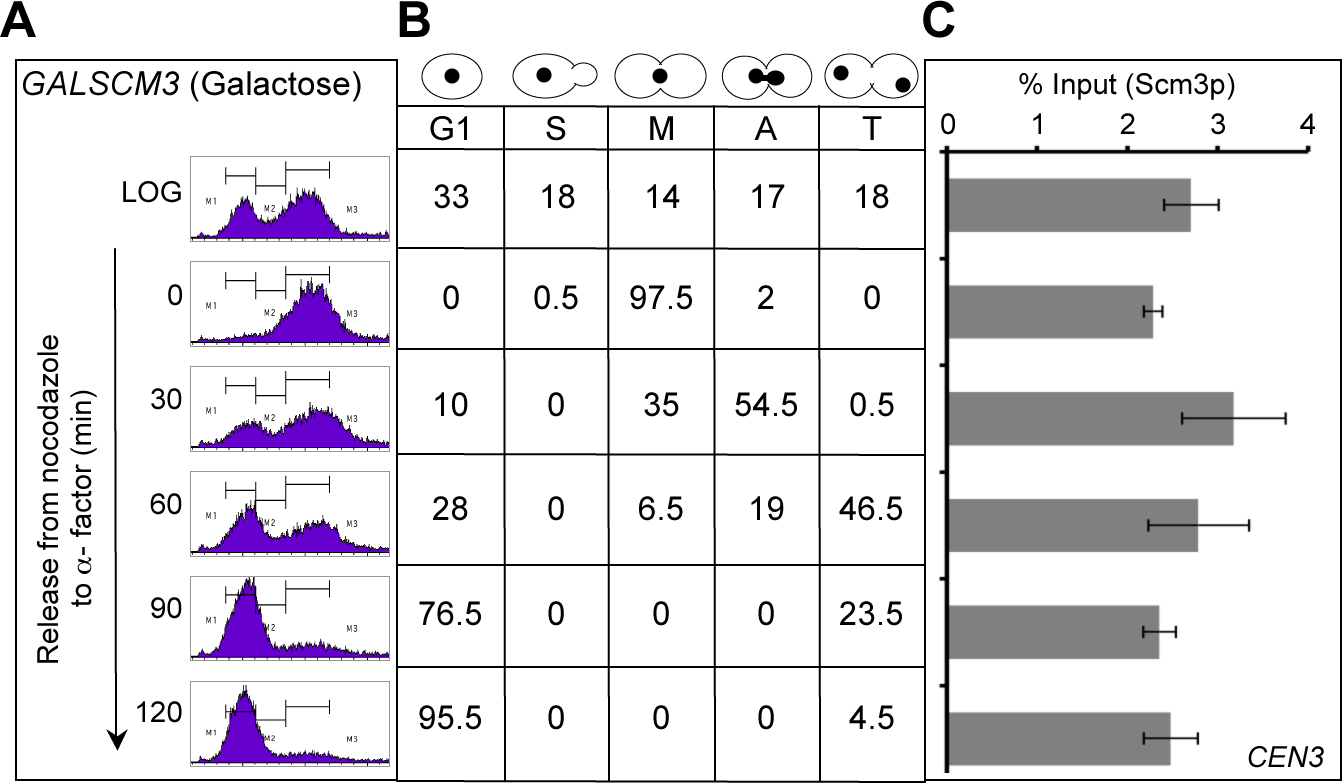

Supplement: Figure S6 — Centromeric enrichment pattern of Scm3p expressed from a GAL1 promoter (GALSCM3) through the cell cycle. Wild-type strain expressing HA tagged Scm3p from GAL1 promoter (RC100) was grown in minimal media with galactose (2%) at 30°C, treated with nocodazole for 2 hours to synchronize cells in G2/M, washed, and released into minimal media containing α-factor. Samples were taken at time points (min) after release from G2/M arrest. (A) DNA content was determined by FACS. (B) Cell cycle stages were determined based on cell morphology and nuclear position by microscopic examination of 200 cells for each time point. (C) Enrichment levels of Scm3p at CEN3. ChIP experiments were done using α-HA (Scm3p), and α-GST (mock) antibodies. The enrichment of Scm3p at CEN3 was determined by qPCR and is shown as % input. Average from at least three independent experiments ± standard error is shown. No significant differences in enrichment were observed among time points. (TIF) [file pgen.1002303.s006.tif]

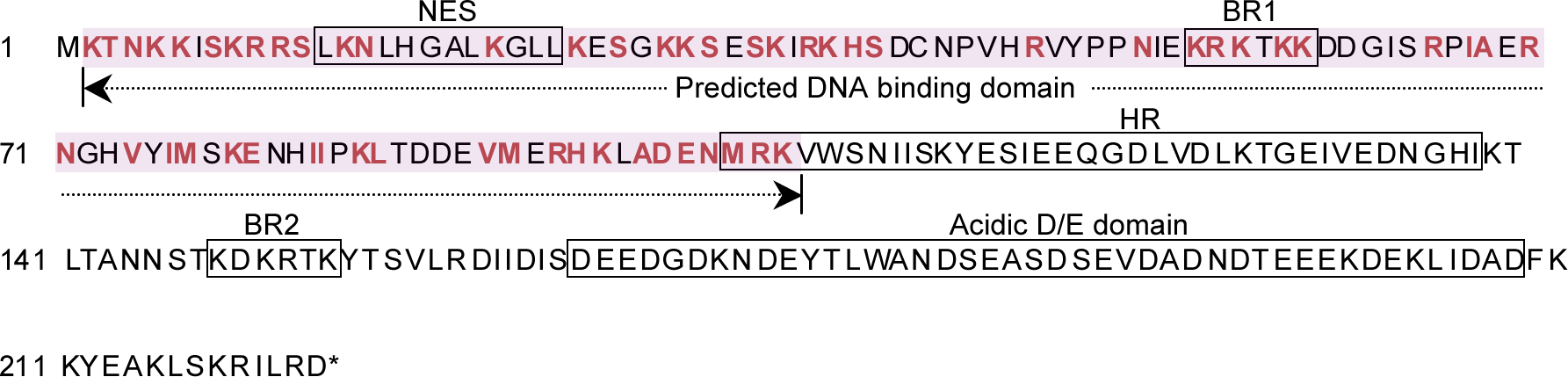

Supplement: Figure S7 — Identification of DNA binding sequences of Scm3p. Predicted DNA binding sequences identified by computational analysis using BindN-RF and MEME software are shown in red and marked with an arrow (amino acids 1-103). Amino acids residues predicted to interact with DNA with high affinity are shown as brown color letters. Symbols, NES = nuclear export signal; HR = Cse4p interacting heptad repeat domain; acidic D/E region, and BR1 and BR2 are basic regions 1 and 2, respectively. (TIF) [file pgen.1002303.s007.tif]
